# Empagliflozin, Linagliptin, and Metformin Differentially Affect Renal PI3K/Akt and MAPK/ERK Signaling Pathways in *db/db* Diabetic Mice

**DOI:** 10.3390/ijms27146483

**Published:** 2026-07-21

**Authors:** Anton I. Korbut, Elizaveta A. Ananishnikova, Nikolai B. Orlov, Nataliya P. Bgatova, Natalia A. Muraleva, Evgenii L. Zavyalov, Vladimir I. Konenkov, Vadim V. Klimontov

**Affiliations:** 1 Research Institute of Clinical and Experimental Lymphology—Branch of the Institute of Cytology and Genetics, Siberian Branch of Russian Academy of Sciences (RICEL—Branch of IC&G SB RAS), Novosibirsk 630117, Russia; korbutai@icgbio.ru (A.I.K.);; 2 Institute of Cytology and Genetics, Siberian Branch of Russian Academy of Sciences (IC&G SB RAS); Novosibirsk 630090, Russia

**Keywords:** type 2 diabetes, chronic kidney disease, PI3K/Akt signaling, MAPK/ERK signaling, empagliflozin, linagliptin, metformin

## Abstract

Accumulating data indicate a role for the dysregulation of the cell cycle, autophagy and apoptosis in diabetic kidney disease. We aimed to evaluate the mediators of the PI3K/Akt and MAPK/ERK signaling pathways in the kidney of *db/db* mice, a model of type 2 diabetes, treated by the SGLT2 inhibitor empagliflozin, the DPP4 inhibitor linagliptin, and metformin. Eight-week-old male *db/db* mice were randomly assigned to treatment by these agents or vehicle for 8 weeks. Age-matched *db/+* mice acted as controls. AMPKα1 and PI3Kp110β were evaluated in the renal cortex and medulla by Western Blot. Phosphorylated forms of principal molecules involved in the PI3K/Akt and MAPK/ERK pathways were assessed by multiplex analysis. *Db/db* mice had decreased PI3Kp110β, increased phospho-PTEN, HSP27 and MEK1 in the renal cortex and medulla, BAD in the renal cortex and decreased phospho-rpS6 in the renal medulla. Empagliflozin prevented the changes in the levels of cortical PI3Kp110β, phospho-MEK1, and medullar phospho-PTEN. Linagliptin restored PI3Kp110β levels. Both agents further decreased medullar phospho-rpS6. Metformin upregulated cortical AMPKα1, medullar PI3Kp110β, phospho-GSK-3α/β and MEK1, and increased phospho-HSP27 in the renal cortex and medulla. The data may provide further explanation of the mechanism underlying the development of diabetic kidney disease, as well as the renal protective effect of anti-diabetic agents.

## 1. Introduction

Diabetes is a driver of chronic kidney disease (CKD) and end-stage renal disease (ESRD) worldwide [1,2]. Studying the mechanisms of development and identifying new molecular targets are essential to define new therapeutic approaches for diabetic kidney disease (DKD). Among the cellular mechanisms of DKD, much attention is paid to impaired proliferation and differentiation of podocytes, mesangial, endothelial and epithelial cells in the kidneys, as well as abnormalities of apoptosis and autophagy in these cells [2,3]. In recent years, significant progress has been made in identifying signaling pathways that regulate the above-mentioned processes. 

The phosphatidylinositol 3-kinases (PI3K)/protein kinase B (Akt) pathway is pivotal for renal pathology. This pathway is involved in apoptosis, epithelial–mesenchymal transition (EMT), inflammation, oxidative stress, and autophagy [4,5]. PI3K is activated by insulin receptor substrate 1 (IRS-1) and is suppressed by phosphatase and tensin homolog (PTEN) [6]. Further, Akt suppresses glycogen synthase kinase 3 α and β (GSK-3α/β), activates mammalian target of rapamycin (mTOR), and deactivates B-cell lymphoma 2 (Bcl-2)-associated death promoter (BAD). The latter stimulates apoptosis and autophagy, while GSK-3 modulates autophagy [7,8]. mTOR leads to activation of ribosomal protein S6 (rpS6), a component of the 40S ribosomal subunit that regulates cell size, proliferation, and glucose homeostasis [9]. PI3K/Akt signaling interacts with the 5′ adenosine monophosphate-activated protein kinase (AMPK) pathway. The primary implication of AMPK signaling in diabetes is the reduction in apoptosis, inflammation, and oxidative stress. In addition, AMPK activates autophagy and downregulates mTOR [8,10,11].

The extracellular signal-regulated kinases 1 and 2 (ERK1/2) are key members of the mitogen-activated protein kinase (MAPK) family. MAPK/ERK signaling is rapidly activated in response to injury and coordinates important pro-regenerative cellular processes, such as cell survival, migration, proliferation, and growth [12]. Dual specificity mitogen-activated protein kinase kinase 1 (MEK1) is a positive regulator of ERK1/2 [13]. Subsequently, ERK1/2 activates the subfamily p90 ribosomal s6 kinase (p90^RSK^) and suppresses cellular tumor antigen p53 [14,15]. Additionally, activation of MAPK/ERK signaling increases phosphorylation of heat shock protein 27 (HSP27) [16]. Both p53 and HSP27 are involved in the regulation of apoptosis and autophagy [8,17,18].

An accumulating body of evidence indicates that dysregulation of PI3K/Akt and MAPK/ERK signaling is involved in the development and progression of DKD. In cell cultures and animal models of diabetes, the activation of PI3K/Akt was associated with endothelial dysfunction, extracellular matrix accumulation, EMT, and tubulointerstitial fibrosis [5,19]. The inhibition of AMPK in podocytes and tubular epithelial cells has been previously reported in diabetes. The inhibition of AMKP in the kidney was associated with obesity-induced lipotoxicity and suppression of glucose transport through tubular cells [20]. In glomeruli, suppression of AMPK is associated with podocytopathy and glomerular fibrosis [21].

Sodium–glucose cotransporter 2 (SGLT2) inhibitors and dipeptidyl peptidase 4 (DPP4) inhibitors opened up new possibilities in the management of DKD. In the Empagliflozin Cardiovascular Outcome Event Trial in Type 2 Diabetes Mellitus Patients (EMPA-REG OUTCOME), and in the Study of Heart and Kidney Protection With Empagliflozin (EMPA-KIDNEY), the SGLT2 inhibitor empagliflozin retarded CKD progression in people with type 2 diabetes (T2D) [22,23]. It was demonstrated that empagliflozin decreases the incidence of macroalbuminuria and retards decline in the estimated glomerular filtration rate (eGFR) in patients with T2D or other CKD-promoting conditions [24,25]. Beneficial cardiac and renal effects were significant for 12 months after the discontinuation of empagliflozin treatment [24,26]. In the Cardiovascular and Renal Microvascular Outcome Study With Linagliptin (CARMELINA) trial, the DPP4 inhibitor linagliptin lowered the risk of development and progression of albuminuria, but had no effect on eGFR decline when compared to the placebo [27,28]. In the treatment of T2D, SGLT2 inhibitors and DPP4 inhibitors are usually combined with metformin [29,30]. This agent is widely accepted to be neutral for renal outcomes [31]. However, some data indicate a lower incidence of ESRD in patients with T2D who received metformin [32].

The protective effect of SGLT2 inhibitors is explained by antihyperglycemic, anti-inflammatory, and anti-hypoxic actions, improving tubule–glomerular feedback and glomerular hemodynamics, as well as indirect impacts on autophagy and apoptosis [33]. The renal-beneficial effects of DPP4 inhibitors are attributed to antihyperglycemic, anti-inflammatory, and anti-oxidant activities [34,35]. The impact of metformin could be associated with a decrease in blood glucose, activation of AMPK, and anti-inflammatory and anti-oxidative effects, as well as mitigation of apoptosis and autophagy abnormalities in the kidney [21,36,37]. The specific role of different signaling pathways in the renal effects of the above-mentioned antihyperglycemic drugs remains to be clarified.

Therefore, we aimed to evaluate the effects of empagliflozin, linagliptin, and metformin on the PI3K/Akt and MAPK/ERK pathways in the kidney of *db/db* mice, a model of T2D.

## 2. Results

### 2.1. Animal Weight and Biochemical Parameters

By the start of the experiment, *db/db* mice had developed obesity and hyperglycemia (Table 1). Throughout the experiment, body weight increased in vehicle-treated, empagliflozin-treated and linagliptin-treated mice (all *p* < 0.05), but not in the metformin group (*p* = 0.42). 

Blood glucose and plasma fructosamine were further elevated in mice that received vehicle and linagliptin (*p* = 0.03 and *p* = 0.02, respectively). Empagliflozin-treated and metformin-treated animals demonstrated no significant changes in these parameters (*p* = 0.23 and *p* = 0.42, respectively).

The baseline plasma levels of creatinine did not differ between the groups of *db/db* and *db/+* mice. However, by the end of the experiment, vehicle-treated *db/db* mice had higher levels of plasma creatinine compared to *db/+* controls (*p* = 0.009). Similarly, higher plasma creatinine was noted in the empagliflozin, linagliptin, and metformin groups in comparison with *db/+* mice (*p* = 0.04, *p* = 0.04, and *p* = 0.009, respectively).

All *db/db* mice were characterized by an elevated urinary albumin-to-creatinine ratio (UACR) by the start of the study (all *p* < 0.001 vs. *db/+* mice). In vehicle- and metformin-treated mice, the UACR remained unchanged (*p* = 0.8 and *p* = 0.9 vs. baseline levels, respectively). However, we noted a decrease in UACR in empagliflozin-treated and linagliptin-treated animals (both *p* < 0.001 vs. baseline levels).

### 2.2. Kidney Weight and Morphology 

We revealed increased kidney weight in vehicle-treated *db/db* mice (*p* = 0.04), but not in the actively treated animals (Table 1). The kidney/body weight ratio was decreased in the empagliflozin and linagliptin groups (both *p* = 0.04 vs. vehicle group).

**Figure 1:**
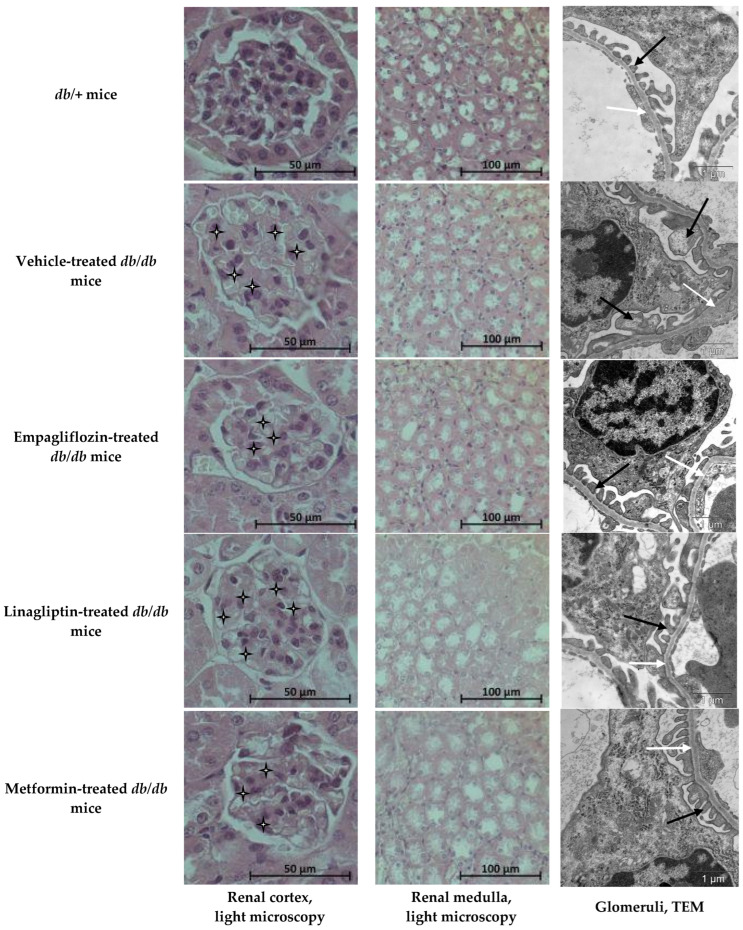
Kidney structure in non-diabetic *db/+* mice and diabetic *db/db* mice treated with vehicle, empagliflozin, linagliptin, or metformin. In *db/db* mice, thickening of the glomerular basement membrane (GBM, white arrows), effacement of the podocyte processes (black arrows), tubular atrophy, and mesangial expansion (white asterisks) were observed. Empagliflozin, linagliptin and metformin decreased mesangial fractional volume and the width of GBM; an increase in the height of collecting duct epithelial cells was observed in empagliflozin-treated *db/db* mice only. Light microscopy (Mayer’s hematoxylin and eosin, H&E), scale bars 50 and 100 μm; transmission electron microscopy (TEM), scale bar 1 μm.

Vehicle-treated *db/db* mice exhibited typical morphological features of diabetic glomerulopathy, including an increase in mesangial fractional volume (MFV), thickening of the glomerular basement membrane (GBM), and podocytopathy. We also observed a decrease in the height of the epithelial cells of the tubules, which may be a sign of their atrophy (Figure 1).

**Figure 2:**
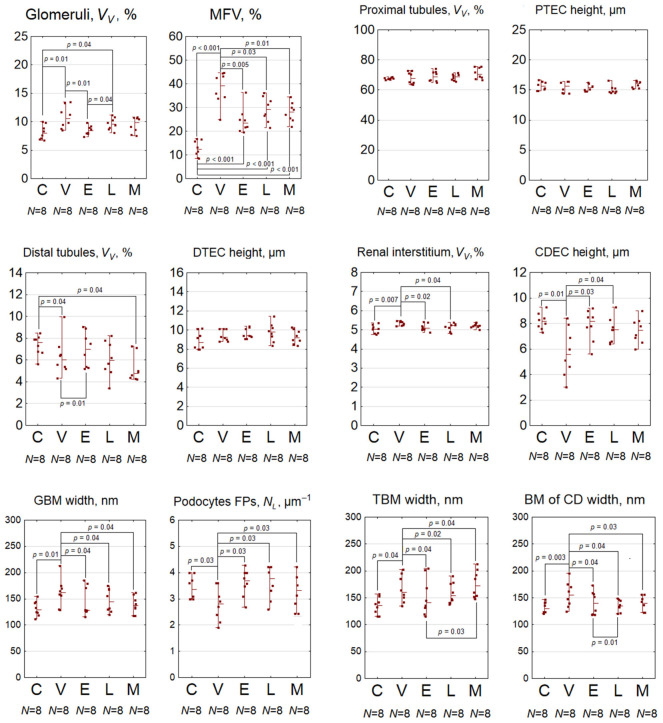
Morphometric parameters in non-diabetic *db/+* mice and diabetic *db/db* mice treated by vehicle, empagliflozin, linagliptin, or metformin. The raw data, averaged for each animal, are presented as points with medians, minimum and maximum values. Differences between groups were assessed using the Mann–Whitney test. Statistically significant differences are shown in the figures. BM, basement membrane; CD, collecting duct; CDEC, collecting duct epithelial cell; DTEC, distal tubular epithelial cell; GBM, glomerular basement membrane width, nm; FPs, foot processes; MFV, mesangial fractional volume; *N_L_*, lineal number density; PTEC, proximal tubular epithelial cell; TBM, tubular basement membrane, nm; *V_V_*, volumetric density; C, control (*db/+* mice); V, vehicle-treated *db/db* mice; E, empagliflozin-treated *db/db* mice; L, linagliptin-treated *db/db* mice; M, metformin-treated *db/db* mice.

These structural changes were assessed by morphometric analysis (Figure 2). In vehicle-treated diabetic *db/db* mice, we found higher glomerular, mesangial and interstitial fractional volumes (*p* = 0.01, *p* < 0.001, and *p* = 0.007, respectively), thickening of GBM, effacement of podocyte foot processes (FPs), thickening of tubular basement membrane (TBM), and lower volume of the distal tubules in the renal cortex (all *p* < 0.05). In addition, these mice demonstrated a decrease in epithelial cell height (*p* = 0.03) and an increase in the width of the basal membrane of collecting duct epithelial cells (CDECs) in the renal medulla compared to control mice (*p* = 0.003).

Diabetic mice treated with empagliflozin, when compared to the vehicle group, had lower glomerular and interstitial *V_V_* (*p* = 0.01 and *p* = 0.04, respectively), TBM thickness in the renal cortex (*p* = 0.04), and width of the basement membrane of collecting ducts in the renal medulla (*p* = 0.008). Similarly, in linagliptin-treated mice, we found lower MFV (*p* = 0.03), GBM and TBM widths (*p* = 0.04 and *p* = 0.02, respectively), and renal interstitium fractional volume (*p* = 0.04) and a higher height of CDECs (*p* = 0.04) compared with the vehicle group. Meanwhile, metformin-treated *db/db* mice demonstrated diminished mesangial expansion (*p* = 0.01), GBM thickening (*p* = 0.04), and CDEC height (*p* = 0.04) when compared with the vehicle-treated group.

Plasma glucose and fructosamine demonstrated positive correlations with MFV (*r* = 0.43, *p* = 0.04 for glucose; *r* = 0.53, *p* = 0.002 for fructosamine) and width of GBM (*r* = 0.57, *p* = 0.01 for glucose; *r* = 0.42, *p* = 0.04 for fructosamine). Both biochemical parameters correlated negatively with the kidney/body weight ratio (*r* = −0.63, *p* < 0.001 for glucose; *r* = −0.53, *p* = 0.002 for fructosamine), podocyte FP *N_L_* (*r* = −0.39, *p* = 0.04 for glucose; *r* = −0.45, *p* = 0.04 for fructosamine), and height of CDECs (*r* = −0.38, *p* = 0.04 for glucose; *r* = −0.43, *p* = 0.02 for fructosamine). 

Plasma creatinine correlated positively with MFV (*r* = 0.45, *p* = 0.04) and the fractional volume of renal interstitium (*r* = 0.46, *p* = 0.03). The UACR positively correlated with MFV (*r* = 0.57, *p* < 0.001) and GBM width (*r* = 0.56, *p* = 0.01). Contrarily, there were inverse correlations between UACR and the podocyte FP *N_L_* (*r* = −0.46, *p* = 0.03) and the height of CDECs (*r* = −0.44, *p* = 0.04).

### 2.3. Markers of Autophagy, Apoptosis and EMT in the Kidney: Results of WB

**Figure 3:**
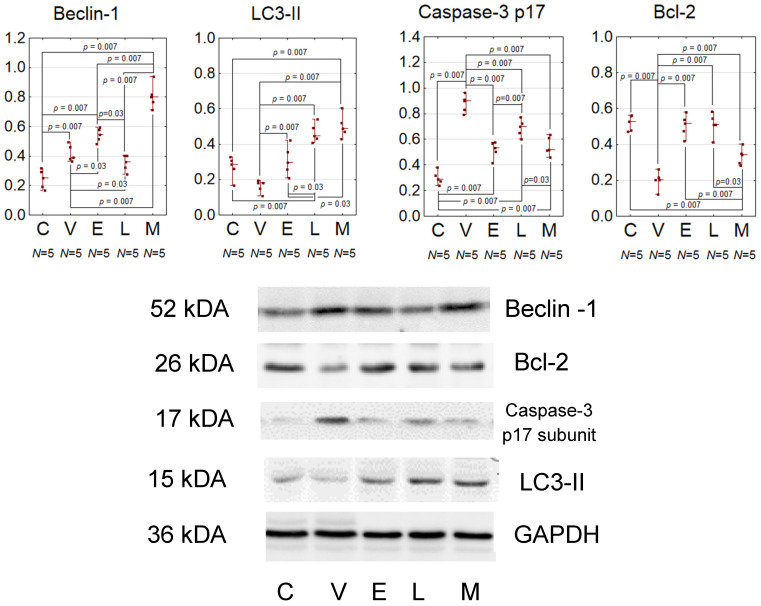
Protein level of Beclin-1, LC3-II, caspase-3 p17 subunit, and Bcl-2 in the renal medulla, arb. units; representative immunoblots of Beclin-1, LC3-II, caspase-3 p17 subunit, Bcl-2, and GAPDH as the control. The raw data, averaged for each animal, are presented as points with medians, minimum and maximum values. Differences between groups were assessed using the Mann–Whitney test. Statistically significant differences are shown in the figures. C, control (*db/+* mice); V, vehicle-treated *db/db* mice; E, empagliflozin-treated *db/db* mice; L, linagliptin-treated *db/db* mice; M, metformin-treated *db/db* mice.

The content of Beclin-1 and caspase-3 p17 subunit in the renal medulla was higher in vehicle-treated *db/db* mice compared to non-diabetic control mice (*p* < 0.01, Figure 3). All treated groups were characterized by higher levels of medullar microtubule-associated proteins 1A/1B light chain 3B (LC3B) lipidated form (LC3-II) and Bcl-2, as well as lower medullar caspase-3 p17 subunit, as compared to the vehicle group (all *p* < 0.05).

The additional elevation in Beclin-1 was significant in the empagliflozin- and metformin-treated groups when compared to vehicle-treated animals (both *p* < 0.05). The highest level of Beclin-1 was noted in the renal medulla of metformin-treated mice (*p* = 0.007 vs. the empagliflozin group), and the highest levels of LC3-II were revealed in linagliptin-treated and metformin-treated mice (*p* = 0.03 vs. the empagliflozin group). The increase in Bcl-2 content was greater in the empagliflozin and linagliptin groups compared with the metformin group (*p* = 0.007 and *p* = 0.03, respectively).

The height of distal tubular epithelial cells (DTECs) correlated negatively with medullar Beclin-1 and caspase-3 p17 subunit (*r* = −0.31, *p* = 0.04 and *r* = −0.46, *p* = 0.03). We also found associations between CDEC height and medullar LC3-II (*r* = 0.44, *p* = 0.03), Bcl-2 (*r* = 0.55, *p* = 0.02), caspase-3 p17 subunit (*r* = −0.49, *p* = 0.03), and Beclin-1 (*r* = −0.44, *p* = 0.03). The baseline body weight was a significant confounder for the medullary expression of LC3-II according to the analysis of covariance results (ANCOVA, *F* = 9.35, *p* = 0.01, *η*^2^ = 0.51, Table S1).

**Figure 4:**
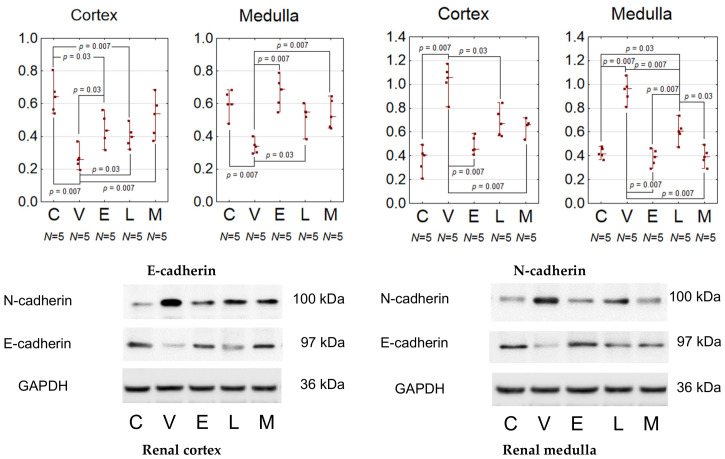
Protein expression of E-cadherin and N-cadherin in the renal cortex and renal medulla, arb. units; representative immunoblots of E- and N-cadherins, and GAPDH as a control. The raw data, averaged for each animal, are presented as points with medians, minimum and maximum values. Differences between groups were assessed using the Mann–Whitney test. Statistically significant differences are shown in the figures. C, control (*db/+* mice); V, vehicle-treated *db/db* mice; E, empagliflozin-treated *db/db* mice; L, linagliptin-treated *db/db* mice; M, metformin-treated *db/db* mice.

Placebo-treated animals demonstrated lower expression of E-cadherin in the renal cortex than *db/+* mice (*p* = 0.007; Figure 4). The cortical expression of E-cadherin was also lower in empagliflozin-treated mice compared to control mice (*p* = 0.03), but it was significantly higher than that in the placebo group (*p* = 0.03). Higher levels of N-cadherin were revealed in the renal cortex of placebo-treated mice as compared to control mice (*p* = 0.007). In the empagliflozin group, there was a decrease in the level of N-cadherin in the renal cortex (*p* = 0.007), which corresponded to that in control animals (*p* = 0.45). Treatment by linagliptin and metformin reversed the DKD-associated changes in the expression of both canderins in the renal cortex (all *p* < 0.05 vs. the vehicle group).

In placebo-treated mice, the content of E-cadherin in the renal medulla was 1.8-fold lower than in the control group (*p* = 0.007; Figure 4). In mice treated with empagliflozin, the medullar E-cadherin level exceeded that in the vehicle group (*p* = 0.007) and corresponded to that of the control group. The expression of N-cadherin in the renal medulla of vehicle-treated mice was higher than in the control group (*p* = 0.007; Figure 4). Empagliflozin-treated mice demonstrated lower medullar levels of N-cadherin compared to the placebo group (*p* = 0.007). Both linagliptin and metformin mitigated the changes in cortical canderins (all *p* < 0.05 vs. the vehicle group).

Cortical E-cadherin positively correlated with the height and *V_V_* of distal tubules (*r* = 0.53, *p* = 0.04 and *r* = 0.58, *p* = 0.02).

### 2.4. AMPKα1 and PI3Kp110β in the Kidney: Results of WB

**Figure 5:**
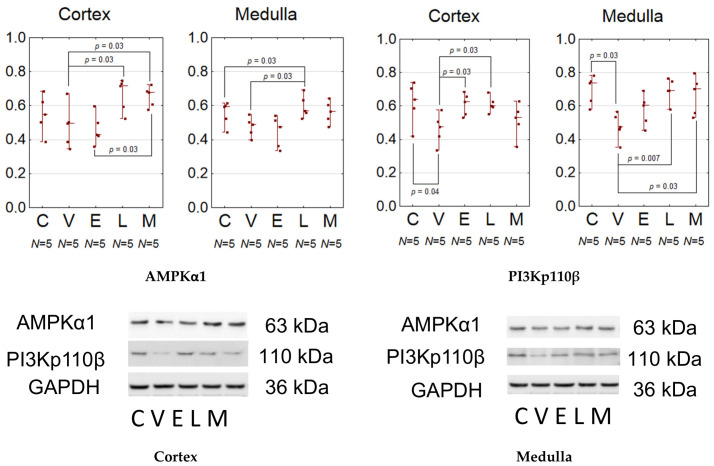
Protein expression of AMPKα1 and PI3Kp110β in the renal cortex and renal medulla, arb. units; representative immunoblots of AMPKα1, PI3p110β and GAPDH as the control. The raw data, averaged for each animal, are presented as points with medians, minimum and maximum values. Differences between groups were assessed using the Mann–Whitney test. Statistically significant differences are shown in the figures. C, control (*db/+* mice); V, vehicle-treated *db/db* mice; E, empagliflozin-treated *db/db* mice; L, linagliptin-treated *db/db* mice; M, metformin-treated *db/db* mice.

The expression of 5′-adenosine monophosphate-activated protein kinase catalytic subunit α1 (AMPKα1) tended to be lower in *db/db* mice that received vehicle than in *db/+* animals (*p* = 0.11, Figure 5). Empagliflozin-treated *db/db* mice did not differ from vehicle-treated ones in the content of AMPKα1. Linagliptin-treated *db/db* mice had higher expressions of AMPKα1 in the renal cortex and medulla compared to vehicle-treated animals (both *p* = 0.03). When compared with vehicle-treated animals, metformin-treated mice demonstrated significantly increased AMPKα1 levels in the renal cortex, but not in the renal medulla (*p* = 0.03 and *p* = 0.22, respectively).

The content of phosphatidylinositol-4,5-bisphosphate 3-kinase catalytic subunit β isoform (phosphoinositide 3-kinase p110β, PI3Kp110β) in the renal cortex and renal medulla was decreased in vehicle-treated *db/db* mice compared with non-diabetic *db/+* control mice (*p* = 0.04 and *p* = 0.03, respectively, Figure 5).

We observed significantly higher expression of PI3Kp110β in the renal cortex of empagliflozin-treated animals compared with the vehicle group (*p* = 0.03), while we did not find this difference in the renal medulla (*p* = 0.89). Linagliptin treatment was associated with higher levels of PI3Kp110β expression in the renal cortex and medulla compared with the vehicle group (*p* = 0.03 and 0.007, respectively). Metformin-treated *db/db* mice had higher levels of PI3Kp110β in the renal medulla but not in the renal cortex compared to vehicle-treated *db/db* mice (*p* = 0.03 and *p* = 0.29, respectively).

We found negative correlations between cortical PI3Kp110β and UACR (*r* = −0.60, *p* = 0.01), MFV (*r* = −0.44, *p* = 0.047), and GBM width (*r* = −0.46, *p* = 0.03). Medullar PI3Kp110β expression correlated positively with the proximal tubular epithelial cell (PTEC) height (r = 0.57, *p* = 0.01). In the renal cortex, the expression of PI3Kp110β correlated negatively with the level of N-cadherin (*r* = −0.44, *p* = 0.01). In the renal medulla, the expression of PI3Kp110β demonstrated a positive correlation with Bcl-2 (*r* = 0.54, *p* = 0.01).

### 2.5. PI3K/Akt Signaling Pathways in the Kidney: The Results of Multiplex Analysis

**Figure 6:**
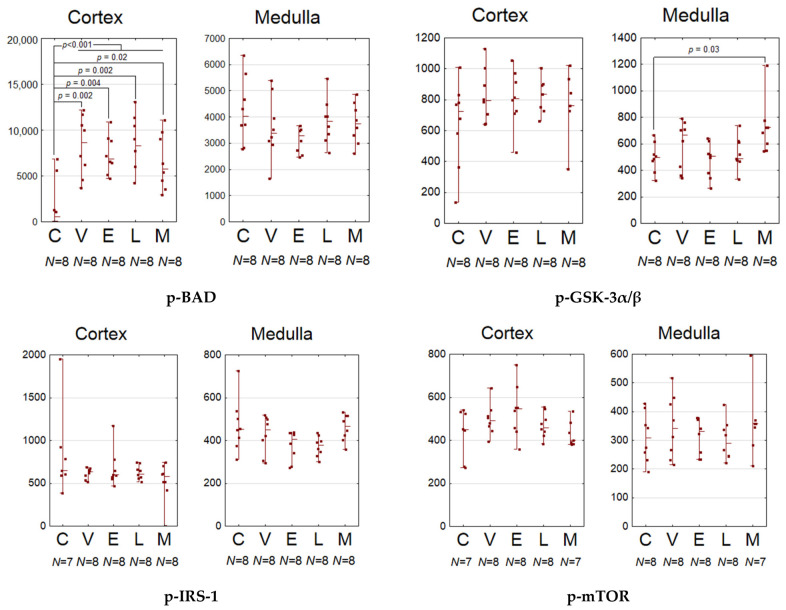

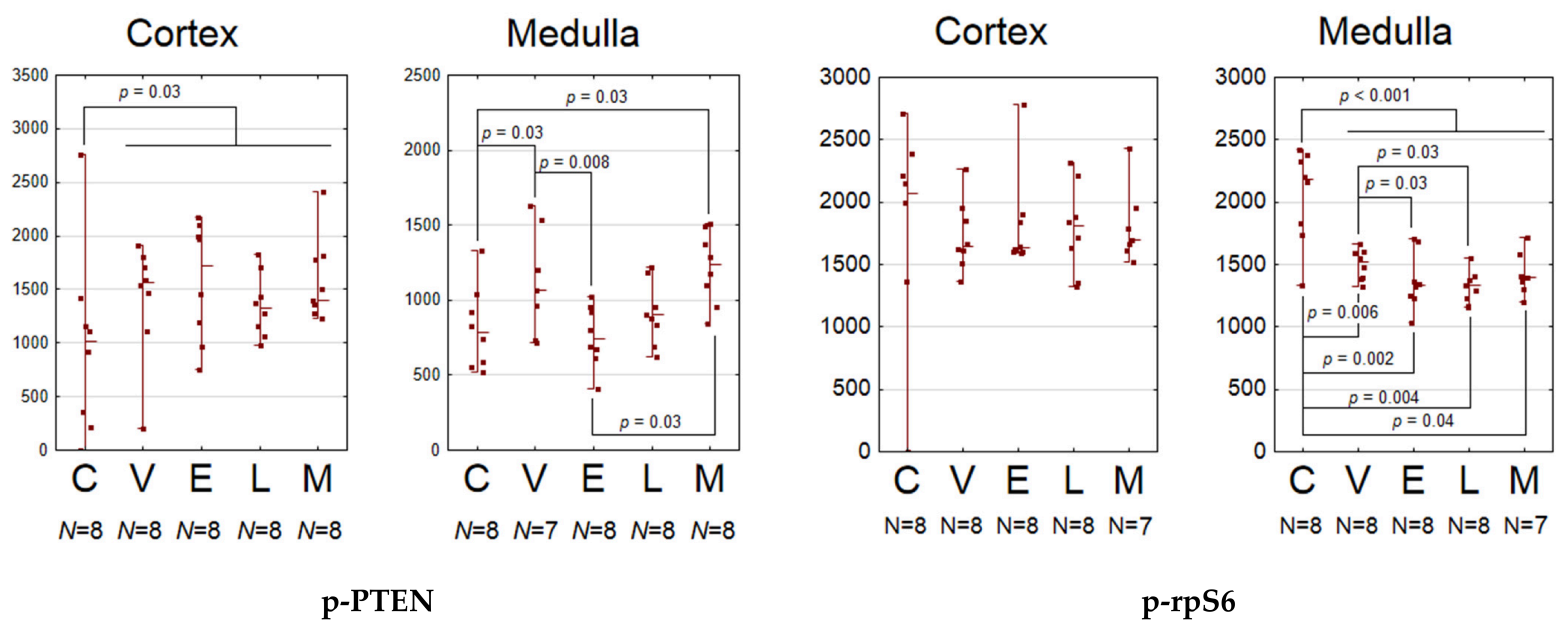
Phosphorylated proteins of the PI3K/Akt signaling pathway in the kidneys of diabetic *db/db* and non-diabetic *db+* mice. Arbitrary units. The raw data, averaged for each animal, are presented as points with medians, minimum and maximum values. Differences between groups were assessed using the Mann–Whitney test. Statistically significant differences are shown in the figures. The following samples were excluded as outliers by Grubbs’ test: phospho-IRS-1, renal cortex, control group (*N* = 1); phospho-mTOR, renal cortex, control group (*N* = 1) and renal cortex and medulla, metformin-treated *db/db* mice (both *N* = 1); phospho-PTEN, renal medulla, vehicle-treated *db/db* mice (*N* = 1); and phospho-rpS6, renal cortex and medulla, metformin-treated *db/db* mice (both *N* = 1). The original sample size was *N* = 8 animals per group. The final sample size after exclusion is shown in the figures. An outlier criterion was specified before examining group differences. C, non-diabetic *db/+* mice (control); E, empagliflozin-treated *db/db* mice; L, linagliptin-treated *db/db* mice; M, metformin-treated *db/db* mice; V, vehicle-treated *db/db* mice.

A higher level of the phosphorylated form of BAD was observed in the renal cortex of *db/db* mice compared to *db/+* control mice (all groups of *db/db* mice vs. *db/+* mice: *p* < 0.001, Figure 6). The differences were also significant for all *db/db* groups (vehicle group: *p* = 0.002; empagliflozin group: *p* = 0.004; linagliptin group: *p* = 0.002; metformin group: *p* = 0.02). However, no differences were found between the three actively treated groups of diabetic animals. We also found no significant differences in phosphorylated BAD levels in the renal medulla between vehicle-treated *db/db* mice and *db/+* control mice, or between actively treated *db/db* mice and the vehicle group (all *p* > 0.05).

Metformin-treated *db/db* mice had significantly elevated levels of phosphorylated GSK-3α/β as compared with *db/+* controls in the renal medulla (*p* = 0.03). We found no other significant differences in this parameter between the groups (all *p* > 0.05).

Phospho-IRS-1 tended to be lower in *db/db* mice as compared to the *db/+* control group in the renal cortex (*p* = 0.096). However, the differences between each specific *db/db* group and *db/+* controls were not significant (*p* = 0.24 for the vehicle group, *p* = 0.15 for the empagliflozin group, *p* = 0.23 for the linagliptin group and *p* = 0.28 for the metformin group). There was no difference in phospho-IRS-1 in the renal medulla.

We noticed no significant differences in the levels of phosphorylated mTOR in the renal cortex and renal medulla between the studied groups of *db/db* and *db/+* mice (all *p* > 0.05).

*Db/db* mice had higher levels of phosphorylated PTEN in the renal cortex compared to *db/+* control mice (*p* = 0.03), with no differences between the diabetic groups. There was an increase in the level of phosphorylated PTEN in the renal medulla of vehicle-treated *db/db* mice (*p* = 0.03). Empagliflozin-treated mice had lower levels of phosphorylated PTEN in the renal medulla compared with vehicle-treated animals (*p* = 0.008). Among actively treated animals, the highest levels of phosphorylated PTEN were revealed in the metformin-treated group (*p* = 0.03 vs. control).

We found no difference between the groups in the levels of phosphorylated rpS6 in the renal cortex (all *p* > 0.05). The content of this molecule was decreased significantly in the renal medulla of *db/db* mice when compared to *db/+* (*p* < 0.001). Empagliflozin-treated and linagliptin-treated mice demonstrated more pronounced lowering of phospho-rpS6 in the renal medulla (*p* < 0.01 vs. controls and *p* < 0.05 vs. placebo); meanwhile, metformin did not significantly change the levels of phosphorylated rpS6 (*p* = 0.04 vs. control group and *p* > 0.05 vs. vehicle).

In *db/db* mice, body weight positively correlated with the content of phosphorylated BAD in the renal cortex and medulla (*r* = 0.49, *p* = 0.009 and *r* = 0.56, *p* = 0.002, respectively). We also found an association between kidney weight and phosphorylated BAD (*r* = 0.39, *p* = 0.03), GSK-3α/β (*r* = 0.57, *p* = 0.02), IRS-1 (*r* = 0.45, *p* = 0.03), and mTOR (*r* = 0.53, *p* = 0.001) in the renal medulla. The height of PTECs correlated negatively with the level of phosphorylated PTEN and IRS-1 in the renal cortex (*r* = −0.61, *p* = 0.003 and *p* = −0.49, *p* = 0.02, respectively). The levels of medullar phosphorylated PTEN and rpS6 were associated with the UACR (*r* = 0.56, *p* = 0.008 and r = 0.43, *p* = 0.049). In the renal medulla, phosphorylated BAD correlated negatively with Bcl-2 (*r* = −0.54, *p* = 0.04). Additionally, medullar phospho-PTEN correlated with E-cadherin and N-cadherin levels (*r* = −0.55, *p* = 0.02 and *r* = 0.5, *p* = 0.04, respectively).

In *db/db* mice, cortical and medullary phospho-BAD were associated with baseline body weight (the results of ANCOVA: *F* = 8.27, *p* = 0.01, *η*^2^ = 0.41 and *F* = 8.01, *p* = 0.02, *η*^2^ = 0.4, respectively, Table S1). The levels of phospho-BAD in the renal medulla were also independently associated with baseline blood glucose (*F* = 8.14, *p* = 0.01, *η*^2^ = 0.4). The contents of cortical phospho-PTEN and medullary phospho-IRS-1 were significantly associated with baseline body weight (*F* = 6.18, *p* = 0.03, *η*^2^ = 0.34 and *F* = 9.8, *p* = 0.009, *η*^2^ = 0.45, respectively).

### 2.6. MAPK/ERK Signaling Pathways in the Kidney: The Results of Multiplex Analysis

**Figure 7:**
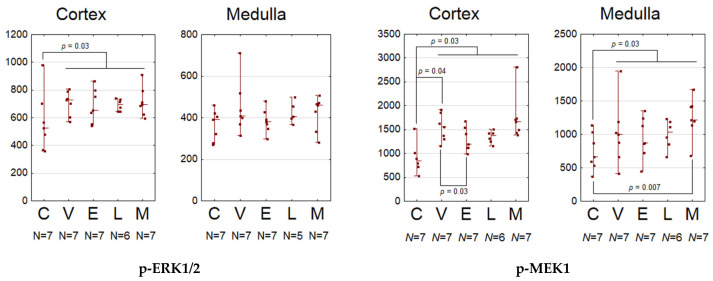

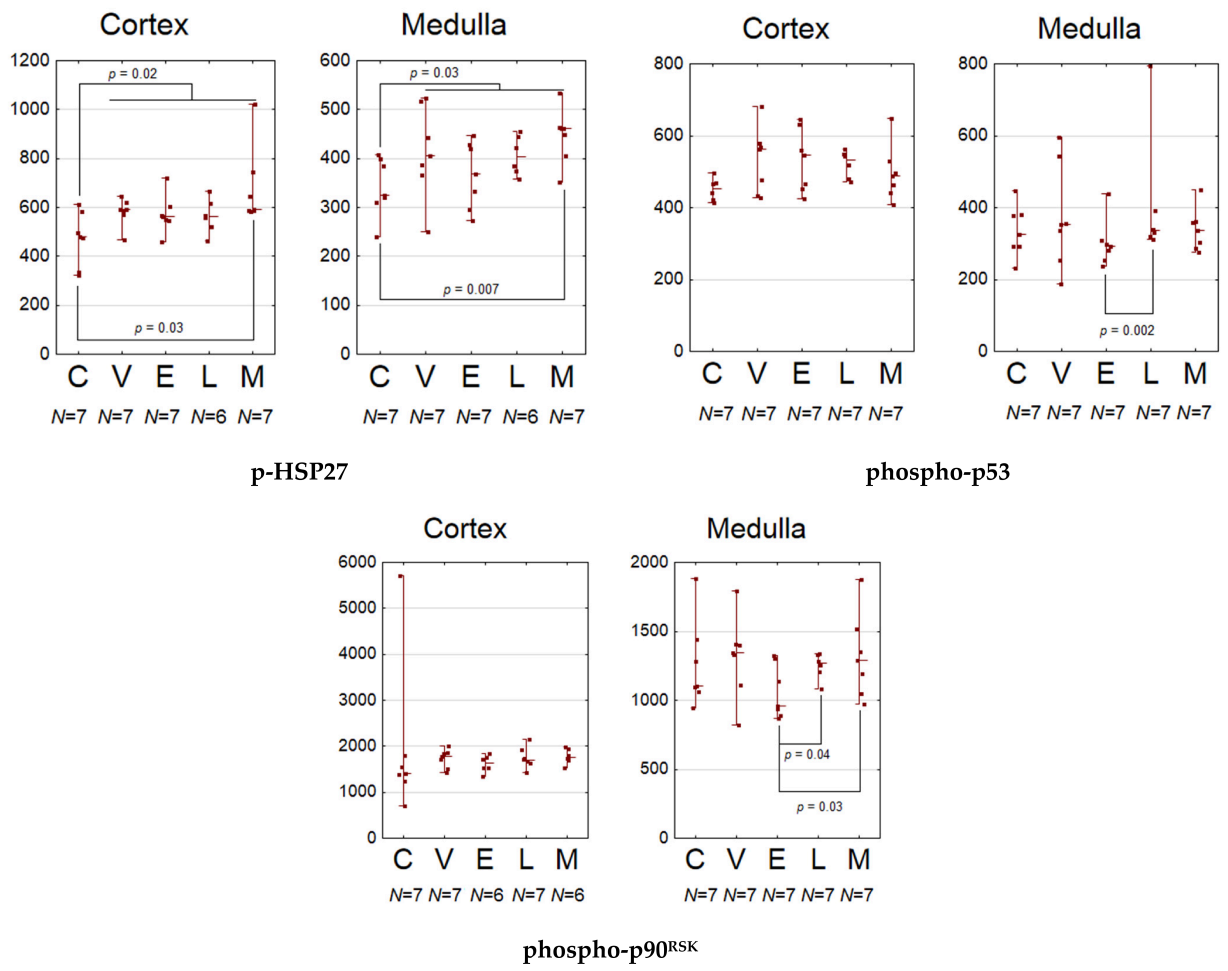
Phosphorylated proteins of the MAPK/ERK signaling pathway in the kidneys of diabetic *db/db* and non-diabetic *db+* mice. Arbitrary units. The raw data, averaged for each animal, are presented as points with medians, minimum and maximum values. Differences between groups were assessed using the Mann–Whitney test. Statistically significant differences are shown in the figures. The following samples were excluded as outliers by Grubbs’ test: phospho-ERK1/2, phospho-MEK1 and phospho-HSP27 in the renal cortex and medulla in linagliptin-treated *db/db* mice (*N* = 2 for phospho-ERK1/2 in the renal medulla and *N* = 1 for other groups); phospho-p90^RSK^, renal cortex, empagliflozin- and metformin-treated *db/db* mice (both *N* = 1). The original sample size was *N* = 7 animals per group. The final sample size after exclusion is shown in the figures. An outlier criterion was specified before examining group differences. C, non-diabetic *db/+* mice (control); E, empagliflozin-treated *db/db* mice; L, linagliptin-treated *db/db* mice; M, metformin-treated *db/db* mice; V, vehicle-treated *db/db* mice. Some samples were excluded as outliers.

A higher level of phosphorylated forms of ERK1/2, MEK1 and HSP27 was found in the renal cortex of *db/db* mice compared to *db/+* control mice (*p* = 0.03, *p* = 0.03, and *p* = 0.02, respectively, Figure 7). The maximum content of phosphorylated MEK1 was revealed in the renal cortex of vehicle-treated *db/db* mice (*p* = 0.04 compared to *db/+* controls). We observed significantly decreased levels of phosphorylated MEK1 in empagliflozin-treated *db/db* mice when compared to the vehicle group (*p* = 0.03). Meanwhile, animals treated with metformin demonstrated higher cortical levels of phosphorylated HSP27 (*p* = 0.03 compared to *db/+* controls).

Similar to the renal cortex, we found increased levels of phosphorylated MEK1 and HSP27 in the renal medulla of *db/db* mice compared to *db/+* (both *p* = 0.03). These changes were more significant in the metformin group (*p* < 0.01 vs. controls).

We found no difference in the level of medullar phosphorylated ERK1/2, p53 and p90^RSK^ between *db/db* and *db/+* mice (all *p* > 0.05). However, we noticed decreased phosphorylated p53 and p90^RSK^ in the renal medulla of empagliflozin-treated *db/db* mice. However, these differences reached statistical significance only when compared with the linagliptin group (p53: *p* = 0.002) or with the linagliptin and metformin groups (p90^RSK^: *p* = 0.04 and *p* = 0.03, respectively), not when compared with the vehicle group (*p* > 0.05 for both).

In *db/db* mice, the height of PTECs negatively correlated with the levels of phosphorylated HSP27 (*r* = −0.63, *p* = 0.003) and p90^RSK^ (*r* = −0.64, *p* = 0.002) in the renal cortex. The *V_V_* of interstitium in the renal cortex negatively correlated with the cortical level of phosphorylated MEK1 (*r* = −0.49, *p* = 0.03). Cortical phosphorylated MEK1 also demonstrated positive relationships with MFV and UACR (*r* = 0.52, *p* = 0.04 and r = 0.48, *p* = 0.03, respectively). We also found an association between kidney weight and expression of phosphorylated HSP27 in the renal medulla (*r* = 0.40, *p* = 0.04). The medullar level of phospho-HSP27 demonstrated associations with EMT markers (E-cadherin: *r* = 0.53, *p* = 0.03; N-cadherin: *r* = −0.63, *p* = 0.007). The expression of N-cadherin positively correlated with phospho-p53 and p90^RSK^ in the renal medulla (r = 0.49, *p* = 0.04 and r = 0.56, *p* = 0.02, respectively). Finally, both phospho-HSP27 and phospho-MEK1 showed positive correlations with LC3-II (*r* = 0.61, *p* = 0.01 and *r* = 0.65, *p* = 0.005, respectively).

We found associations between the cortical phospho-MEK1 and baseline body weight in *db/db* mice (the results of ANCOVA: *F* = 5.64, *p* = 0.04, *η*^2^ = 0.36, Table S1). The levels of phospho-HSP27 in the renal cortex were also independently associated with baseline body weight and blood glucose (*F* = 22.4, *p* = 0.0008, *η*^2^ = 0.69 and *F* = 19.9, *p* = 0.001, *η*^2^ = 0.67, respectively).

## 3. Discussion

The results of our study indicate that the development of diabetic nephropathy in *db/db* mice, a model of T2D, is associated with a dysregulation of the PI3K/Akt and MAPK/ERK signaling pathways in the renal cortex and medulla. This dysregulation is associated with abnormal expression of some markers of autophagy, apoptosis, and EMT. We also demonstrate differences in the effects of the SGLT2 inhibitor empagliflozin, the DPP4 inhibitor linagliptin, and metformin on renal PI3K/Akt and MAPK/ERK signaling.

### 3.1. Effects of Empagliflozin, Linagliptin, and Metformin on Body Weight, Blood Glucose, Renal Function and Structural Parameters in db/db Mice

In the experimental model of obesity and T2D we used, the three anti-diabetic drugs studied had different effects on metabolic parameters and body weight. Specifically, metformin-treated and empagliflozin-treated *db/db* mice demonstrated lower blood glucose as well as plasma fructosamine levels, while linagliptin had no effect on hyperglycemia. These findings are generally consistent with literature data. The antihyperglycemic potential of metformin and empagliflozin in *db/db* mice was demonstrated previously [38,39,40,41]. However, no glucose-lowering effect of linagliptin was observed in this model [42,43]. In humans, DPP4 inhibitors demonstrate modest antihyperglycemic activity and are used in patients with mild or moderate hyperglycemia [30,44]. On the contrary, *db/db* mice demonstrate severe hyperglycemia due to leptin receptor deficiency [45,46]. Therefore, we can consider the lack of glucose-lowering effects of the DPP4 inhibitor in our study to be expected.

We observed no influence of empagliflozin or linagliptin on the body weight of *db/db* mice. At the same time, metformin prevented further weight gain in these animals. In previous studies, it was noted that DPP4 inhibitors have no significant effect on body weight in *db/db* mice [47,48]. The results for SGLT2 inhibitors are quite contradictory. Some studies reported slower body weight gain in *db/db* mice during treatment by empagliflozin [48,49,50,51]. Other studies have found no effect of empagliflozin on animal weight in this model [52,53]. Moreover, an increase in body weight was reported in *db/db* mice treated with empagliflozin [40,54,55,56]. In clinical trials, SGLT2 inhibitors have been shown to reduce body weight in humans [57]. The exact mechanism behind the effect of empagliflozin on body weight in *db/db* mice needs future research.

We did not find any differences in creatinine levels between the groups of animals treated with empagliflozin, linagliptin, and metformin. This may be due to the timing of the experiment: a decline in kidney function in *db/db* mice usually occurs after the age of 16 weeks [58,59]. At the same time, we observed a significant antialbuminuric effect of empagliflozin and linagliptin, but not metformin, in *db/db* mice in our experiment, which is consistent with the results of clinical studies [24,28,32].

We observed protective effects of empagliflozin, linagliptin, and metformin on the renal structural changes in *db/db* mice. Specifically, treatment by empagliflozin was associated with mitigation of mesangial expansion, signs of podocytopathy and tubular atrophy in *db/db* mice. The beneficial effects of empagliflozin on glomerular and tubular cells were described previously in *db/db* mice [60,61]. Similar effects have been demonstrated in this model under linagliptin or metformin [62,63].

Taking into account the key roles of the PI3K/Akt and MAPK/ERK pathways in the regulation of autophagy, apoptosis and EMT, we determined the content of some markers of these processes in the renal medulla by WB. We found elevated levels of Beclin-1 (but not LC3-II) and caspase-3 p17 subunit and lower levels of Bcl-2 in *db/db* mice compared to non-diabetic control mice. Among these molecules, Beclin-1 is considered to be a key regulator of autophagy. The protein interacts with either Bcl-2 or PI3K class III, regulating autophagy and cell death [64]. Bcl-2 is considered to be an anti-apoptotic protein [64], and the cleaved, active form of caspase-3 (p17) is an enzyme known for its crucial role in apoptosis [65]. Although we did not study the structural features of apoptosis and autophagy flux in our animals, these WB data may serve as indirect indicators of changes in the discussed processes.

In animals treated with empagliflozin, linagliptin, or metformin, we observed increased LC3-II levels. It should be noted that two bands (LC3-I and LC3-II, cytosolic and lipidated forms of LC3B, respectively) are typically seen in WB performed using an anti-LC3B antibody. In our blots, we observed a single band that migrated at approximately 15 kDa, which corresponds to LC3-II. The signal for LC3-I was weak under our experimental conditions, likely due to its conversion to LC3-II. Therefore, the band that we observed with a high probability corresponds to LC3-II, a marker of autophagosomes. An increase in LC3-II may reflect either increased autophagosome formation or impaired autophagosome degradation [8]. Future studies examining autophagic flux are warranted to elucidate these findings.

A decreased caspase-3 p17 subunit and increased Bcl-2 content were observed in actively treated mice. Administration of empagliflozin or metformin was also associated with an elevation in Beclin-1. These data can be interpreted, with some caution, as changes in apoptotic, anti-apoptotic, and autophagy-related signaling under the influence of the drugs studied.

We found decreased E-cadherin and elevated N-cadherin levels in the renal cortex and medulla of *db/db* mice. In our experiment, these shifts were associated with the signs of tubular atrophy. The association of cadherin expression changes with EMT in DKD was also reported [66]. SGLT2 inhibitors can reverse the changes in cadherin expression and suppress EMT and diabetes-induced renal fibrosis [33,66]. Treatment by linagliptin was found to mitigate transforming growth factor β1 (TGFβ1)-mediated EMT in tacrolimus-induced renal interstitial fibrosis in rats [67]. The suppressive effect of metformin on EMT in renal cells was also described [68,69,70].

### 3.2. Effects of Empagliflozin, Linagliptin, and Metformin on PI3K/Akt Signaling in the Kidney in db/db Mice

We found downregulation of PI3Kp110β in the renal cortex and medulla of *db/db* mice. Hyperglycemia and oxidative stress are considered to be the primary factors dysregulating PI3K/Akt signaling in diabetes [5]. The disruption of PI3K/Akt signaling may promote insulin resistance in renal cells [19]. It was estimated that PI3Kp110β promotes autophagy in mammalian cells, including kidney cell culture [8,71]. In its turn, suppression of autophagy is involved in the development of DKD [69,70,71,72,73]. We revealed an increased cortical level of PI3Kp110β in empagliflozin-treated and linagliptin-treated *db/db* mice. We also noted increased levels of PI3Kp110β in linagliptin-treated and metformin-treated animals in the renal medulla. It was demonstrated that empagliflozin and linagliptin reactivate autophagy in the renal cortex, glomeruli, and podocytes in *db/db* mice [74]. Likewise, the reactivation of autophagy in tubular cells was also found in metformin-treated *db/db* mice [75]. Therefore, further research is needed to find out if the reactivation of autophagy in the renal cortex and medulla is associated with upregulation of PI3Kp110β under empagliflozin, linagliptin and metformin treatments.

In our study, both metformin and linagliptin enhanced the levels of AMPKα1 in the renal cortex and medulla. Metformin is a well-known activator of AMPK [36,76]. It was demonstrated that metformin alleviates podocyte loss, apoptosis of mesangial cells, and tubular cell senescence through AMPK-mediated signaling pathways in DKD [77]. On the other hand, it was reported that metformin-induced AMPK upregulation is associated with activation of apoptosis in renal medullary interstitial cells [78]. Modulation of AMPK activity is also considered to be an effect of DPP4 inhibitors [79]. In NRK-52E cells (rat renal proximal tubule cell line), linagliptin attenuated ROS production by activating the AMPK pathway and decreased pro-inflammatory cytokines, such as tumor necrosis factor α and interleukin 1β [80]. In a rat models of T2D, linagliptin enhanced renal expressions of phospho-AMPK/AMPK, improved proteinuria, and alleviated renal ultrastructural changes and fibrosis [81]. In our study, we found no association of AMPKα1 with autophagy, apoptosis, and EMT markers. Therefore, the contribution of AMPKα1 to the renal protective effects of anti-diabetic drugs remains to be clarified.

We found significantly elevated levels of phosphorylated forms of the pro-apoptotic protein BAD in the renal cortex in *db/db* mice. It is well known that DKD is associated with activation of apoptosis in renal cells [2,3]. On the other hand, BAD acts as an activator of autophagy [8]. We were unable to demonstrate any significant effect of the three studied drugs on the levels of this regulator.

We observed a higher level of phosphorylated PTEN in the renal cortex and medulla of *db/db* mice. PTEN is a suppressor of the PI3K/Akt/mTOR pathway and a crucial regulator of cell death, autophagy, and apoptosis [6]. In patients with T2D, elevated urinary PTEN is associated with CKD [82]. Phosphorylation at Ser380 generally inactivates PTEN, reduces the ability of PTEN to inhibit the PI3K/Akt pathway, and promotes cell survival [83]. In non-diabetic mice, *PTEN* knockout in tubular cells resulted in renal hypertrophy [84]. We found that the levels of phosphorylated BAD and PTEN in the renal cortex correlated negatively with the height of PTECs in *db/db* mice. Moreover, we found associations of phospho-PTEN with the markers of EMT (E-cadherin and N-cadherin) in the renal medulla of *db/db* mice. Therefore, we can speculate that increased levels of phosphorylated BAD and PTEN may contribute to tubular atrophy and EMT in this model of diabetes. Empagliflozin treatment only was associated with lower medullar levels of phosphorylated PTEN.

The exact effects of phosphorylation and dephosphorylation of PTEN are highly context-dependent [85]. Generally, the phosphorylation of Ser380 deactivates PTEN and the diminishing capacity of PTEN to inhibit the PI3K/Akt pathway [83]. In our study, increased medullar level of phosphorylated PTEN were associated with albuminuria and abnormalities in N-cadherin and E-cadherin expression. It can be assumed that activation of PTEN through decreased phosphorylation under treatment by empagliflozin is associated with the mitigation of albuminuria and EMT.

We found a decreased level of phosphorylated rpS6 in the renal medulla of *db/db* mice. rpS6 is involved in the regulation of glucose homeostasis, insulin sensitivity, body mass and energy balance, as well as cell size [86]. Phosphorylation of rpS6 at serine 235/236 is a crucial step in activating protein synthesis, especially for mRNAs encoding cell growth and proliferation proteins [87]. Phosphorylation of rpS6 was associated with renal hypertrophy in a unilateral nephrectomy model [88]. Another study demonstrated that the blocking of rpS6 phosphorylation exacerbates kidney growth in a non-diabetic model [89]. In our study, we found no correlations between the levels of rpS6 in the renal cortex or medulla and kidney weight in *db/db* mice. However, medullar phospho-rpS6 was associated with tubular atrophy and elevated N-cadherin expression.

The treatment by empagliflozin or linagliptin was associated with decreased levels of phosphorylated rpS6 in the renal medulla of *db/db* mice. However, genetic knockout and pharmacological inhibition of rpS6 phosphorylation have been demonstrated to prevent podocyte hypertrophy and mitigate focal segmental glomerulosclerosis in patients and in a rat model [90]. In a model of diabetes, attenuated phosphorylation of rpS6 was found to be associated with the antifibrotic activity of resveratrol in heart and skeletal muscles [91].

Decreased phosphorylation of rpS6 may be due to the influence of different factors, such as energy depletion [92]. A shift in energy metabolism towards an energy-saving state is a characteristic feature of the action of SGLT2 inhibitors [93]. We may suppose that downregulation of rpS6 reflects the change in the energy balance in the renal medulla. Notably, although *db/db* mice already exhibited reduced medullary phospho-rpS6 compared to non-diabetic control mice, treatment by empagliflozin or linagliptin further lowered its levels. This seemingly paradoxical effect may be explained by a compensatory mechanism: in untreated diabetes, residual rpS6 phosphorylation might sustain maladaptive hypertrophy, whereas additional suppression by these drugs could shift cellular metabolism toward a more protective, energy-conserving state. At the same time, phospho-rpS6 is considered to be a marker of impaired protein synthesis and cellular stress [9]. Therefore, the precise role of this regulator in the development of DKD and its modulation by empagliflozin and linagliptin deserves further study.

### 3.3. Effects of Empagliflozin, Linagliptin, and Metformin on the MAPK/ERK Pathway in Kidneys of db/db Mice 

MAPK/ERK signaling plays a role in the regulation of renal development and differentiation [94]. Phosphorylation at Thr202/Tyr204 and Thr185/Tyr187 is an activating post-translational modification of ERK1/2 [95]. Similarly, phosphorylation at Ser78 and phosphorylation at Ser217/Ser221 activate HSP27 and MEK1, respectively [96,97]. Activation of MEK/ERK signaling in mesangial cells has been shown to be involved in mesangial expansion in diabetes [98]. Finally, MEK/ERK signaling is considered to contribute to the suppression of renal autophagy in a rat model of diabetes [99]. The suppression of autophagy in the kidneys of *db/db* mice, as well as the ability of empagliflozin to reactivate renal autophagy, were demonstrated in previous studies [74,100].

In this study, we found an elevated expression of phosphorylated forms of ERK-1/2, HSP27, and MEK1 in the renal cortex of *db/db* mice. We also revealed higher levels of phosphorylated HSP27 and MEK1 in the renal medulla. Treatment by empagliflozin was associated with lower cortical levels of phosphorylated MEK1.

Elevated serum and urinary HSP27 are considered to be specific and early markers of DKD [101,102]. In the kidney, HSP27 is localized in podocytes, tubular epithelial cells and endotheliocytes [103]. In our study, an increased content of phosphorylated HSP27 in the renal cortex was associated with tubular atrophy, while increased phosphorylated HSP27 in the renal medulla was associated with kidney hypertrophy.

In our experiment, empagliflozin-treated animals demonstrated the lowest levels of phosphorylated p53 and p90^RSK^ in the renal medulla. p53 is a transcription factor that suppresses cell proliferation [104]. Elevated phosphorylation of p53 was found to play a role in the development and progression of CKD due to increased expression of TGFβ1 [105]. Targeting p53 is a promising approach for organ fibrosis therapy [106]. p90^RSK^ belongs to a protein family that regulates cell motility and survival. An elevated expression of p90^RSK^ is associated with renal fibrosis and DKD [17,18]. The levels of phosphorylated p90^RSK^ in the renal cortex were negatively associated with tubular cell atrophy. Interestingly, suppression of p90^RKS^ in models of lung fibrosis and breast cancer was associated with suppression of EMT [107,108]. EMT is also known as a process involved in the development and progression of DKD [109]. In *db/db* mice, treatment by empagliflozin was associated with less pronounced changes in the renal expression of cadherins, which are involved in EMT [110]. Thus, decreased p53 and p90^RSK^ under empagliflozin may be a possible mechanism underlying the suppression of renal fibrosis and EMT in the kidney.

### 3.4. Methodological Remarks and the Study Limitations

In this study, we assessed a panel of key molecules involved in PI3K/Akt and MAPK/ERK signaling. Both signaling pathways are complex and include a number of molecules interacting with each other and forming cascades and networks [111,112,113]. In such cases, the measurement of individual regulators may not provide a comprehensive view of the molecular changes [114]. We assessed the phosphorylated forms of the proteins, taking into account that this post-translational modification regulates the activity of signal transduction [115]. In order to avoid pseudoreplication, we averaged the technical replicates of measurements for several observational units, with the animals as biological and experimental units, and used these averages for further statistical analysis of morphometry results, as recommended [116].

The limitations of the study include the small sample size, the absence of drug-treated non-diabetic control groups, the lack of blinding in some experimental procedures, and the use of a single terminal time point. The small sample size limits the representativeness of the results. Although the animals were randomly allocated, baseline imbalances between the groups in body weight and blood glucose could introduce residual confounding. The absence of a non-diabetic control group treated with the studied drugs limits our ability to dissociate drug-specific effects from antihyperglycemic and body weight-lowering effects. A single time point does not provide any insight into the dynamics of molecular changes.

The changes in AMPKα1 and PI3Kp110β levels were only validated by WB. We used glyceraldehyde 3-phosphate dehydrogenase (GAPDH) as a loading control in the WB study. As GAPDH may be affected by the metabolic alterations associated with diabetes [117], its use as a loading control could be another possible limitation. Accordingly, we compared GAPDH expression stability with that of β-actin, an alternative housekeeping protein, under our experimental conditions. We further tested our samples using total protein stained with Ponceau S for normalization and demonstrated identical patterns of protein changes.

In this study, we focused predominantly on examining alterations in the signaling pathways, without directly assessing proliferation, apoptosis, autophagy, and EMT in the kidney. In an attempt to overcome this limitation, at least partially, we studied the expression of a number of molecules involved in the regulation of these processes in the renal medulla. However, the limited set of markers and the lack of renal cortex data did not allow us to answer the question about the causal role of the changes in the signaling pathways. The results do not provide a clear assessment of the intensity of the above-mentioned cellular processes. In particular, the autophagic flux was not measured, and the present results cannot distinguish increased autophagosome formation from reduced autophagosome clearance. Therefore, further research is needed for this.

## 4. Materials and Methods

### 4.1. Design

**Figure 8:**
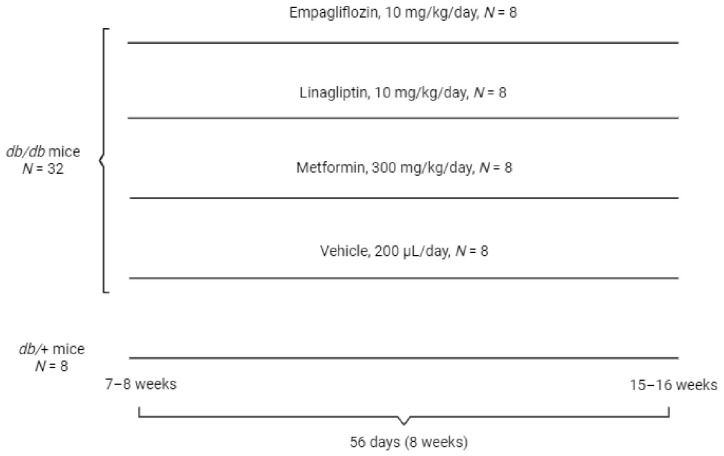
Flowchart of the study. The experiment used 7–8-week-old non-diabetic *db/+* mice and diabetic *db/db* mice. Diabetic mice were randomly assigned to treatment by a vehicle, empagliflozin, linagliptin, or metformin. The total duration of the experiment was 8 weeks.

The design of the study is presented in Figure 8. We used specific pathogen-free male *db/db* mice (BKS.Cg-Dock7^m^+/+Lepr^db^/Icg) obtained from the Center for Genetic Resources of Laboratory Animals at ICG SB RAS (Novosibirsk, Russia) for the experiment. Due to a mutation in the gene of the leptin receptor, these mice become hyperphagic early in their life and develop marked obesity, hyperglycemia, and hyperinsulinemia [45,46]. Therefore, *db/db* is a widely used model of obesity and T2D [118]. The animals demonstrate structural changes in the kidneys that are similar to those in humans with diabetes [59,60,119]. The model is characterized by the slow-to-moderate progression of nephropathy [120]. Sibling non-diabetic *db/+* male mice were used as the control.

The animals were housed in a carefully controlled setting, with a consistent 12-h day/night cycle, a stable temperature of 23 ± 2 °C, and a humidity level of 45 ± 10%. The mice, aged three weeks, were separated from their mothers and placed in individually ventilated cages (OptiMICE, Animal Care Systems, Centennial, CO, USA), with two to three mice per cage. They had unrestricted access to food (Ssniff, Soest, Germany) and water.

Diabetic mice were assigned by simple randomization method to receive empagliflozin (Boehringer Ingelheim, Ingelheim, Germany; 10 mg/kg), linagliptin (Boehringer Inhelheim, Ingelheim, Germany; 10 mg/kg), metformin (Ozon Pharmaceuticals, Zhigulevsk, Samara Region, Russia; 300 mg/kg), or saline (200 µL). The dosages of empagliflozin, linagliptin, and metformin were selected according to previous investigations [121,122,123]. The medicines were diluted in 200 µL of saline. Intragastric administration was performed once a day with 20 G × 38 mm curved oral dosing needles (pk/3, VetTech Solutions Ltd., Congleton, UK). All animals were treated for 56 days from 7–8 to 15–16 weeks of life by the investigator, who was aware of the group allocation. Eight animals were enrolled in each group.

The animals were weighed weekly. The animals were starved overnight with access to water before blood collection. Blood sampling to determine glucose concentration was carried out from the tail. Blood samples for the assessment of other biochemistry parameters (150–200 μL) were collected from the retro-orbital venous sinus at weeks 7–8, 11–12 and 15–16 of life, using a glass Pasteur pipette previously wetted by heparin 5000 IU/mL. To obtain plasma, the blood samples were centrifuged at 3000× *g* and +4 °C for 15 min after collection. Urine samples were collected at week 7–8 and week 15–16 of age, prior to the blood sampling. After collection, the urine samples were centrifuged at 3000× *g* and +4 °C for 15 min; the supernatant was separated and used for the following investigation. The plasma and urine samples were labeled, frozen immediately, and stored at −80 °C for biochemistry investigations.

All animals were decapitated at week 15–16. To assess renal hypertrophy, kidneys were weighed and adjusted to body weight. Kidney samples were obtained for histological and ultrastructural examination and assessment of signaling pathways by multiplex analysis and WB. The researchers who performed biochemical investigations, ELISA, microscopy, WB, and multiplex bead array assays were not aware of the group allocation.

All invasive procedures with animals (blood collection and harvesting) were performed under anesthesia with isoflurane (Laboratories Karizoo, S.A., Caldes de Montbui, Barcelona, Spain) and Gas Anesthesia System Ugo Basile (Gemonio, Italy, catalog 21210). The study had no human endpoint. No adverse events were noticed during the experiment.

### 4.2. Biochemistry and ELISA

The fasting blood glucose level was measured by a Contour TS blood glucose meter (Bayer, Leverkusen, North Rhine-Westphalia, Germany) immediately after blood sampling.

Plasma and urinary creatinine concentrations were estimated by applying the Jaffe methods (DiaS, Moscow, Russia). Plasma fructosamine and urinary albumin concentrations were assessed by an enzyme-linked immunosorbent assay (ELISA) with commercially available kits (BlueGen, Shanghai, China, lots E03F0411 and E04A0028, respectively). The concentrations of urinary albumin were adjusted to excreted creatinine.

Biochemical investigations were performed on each animal. Due to the small volume of biological samples available for analysis, biochemical studies were carried out in one technical replicate. The results were used as the experimental units in the following statistical analysis.

### 4.3. Microscopy and Morphometry

#### 4.3.1. Light Microscopy

Kidney samples from each animal (*N* = 8 per group) were fixed in 10% formalin (pH = 7.4) for 48 h, dehydrated in alcohol at increasing concentrations and embedded in Histomix material (BioVitrum, Saint Petersburg, Russia) using a Thermo Scientific™ Citadel 2000 Tissue Processor and Thermo Scientific™ HistoStar™ Embedding Workstation (Fisher Scientific, Pittsburgh, UK). Then, 5 µm thick sections were prepared on a microtome LEICA RM2155 (Leica Microsystems GmbH, Wetzlar, Germany) and were stained with Mayer’s hematoxylin and eosin (H&E). The sections were counterstained, dehydrated, and mounted.

Light optical studies were performed with an Axioskop 2 plus microscope (Zeiss, Oberkochen, Germany). The manufacturer’s software Axiovision 2.0 (Zeiss, Oberkochen, Germany) was used to acquire images with a size of 1300 × 1300 pixels. Ten random non-overlapping areas of the renal cortex on one or two kidney sections were photographed to provide the pictures for each animal (*N* = 8 per group) at a primary magnification of ×100. These images were used to assess the *V_V_* of glomeruli, proximal and distal tubules.

Images of 15 non-overlapping areas of the renal cortex and medulla of each animal (*N* = 8 per group, one kidney section per animal) were obtained and investigated at a final magnification of ×400. At least 10 renal corpuscles with vascular and urinary poles from the inner, mid, and outer renal cortex, 10 proximal tubules, 10 distal tubules, and 10 collecting ducts per animal (*N* = 8 per group) were selected for the assessment of MFV and the height of PTECs, DTECs, and CDECs, respectively. Tangentially sectioned, incomplete, folded, artifact-containing, or stain-precipitate-containing glomeruli were not used for the assessment of MFV.

#### 4.3.2. Transmission Electron Microscopy

Renal cortex and medulla samples from each animal (*N* = 8 per group) were fixed in a 4% solution of paraformaldehyde with 0.1 M phosphate buffer (pH = 7.4), followed by 1% OsO_4_. The samples were then dehydrated and embedded in EPON epoxy resin. The 70–100 nm kidney sections (two or three per animal) were contrasted with aqueous uranyl acetate solution and lead citrate and were studied with a JEM-1400 electron microscope (JEOL, Akishima, Tokyo, Japan). The number of sections for quantification of FPs was determined based on the data of Shi et al. [124].

To estimate the *N_L_* of podocyte FPs, 10 non-overlapping images of the cross-sectioned glomerular barrier were obtained at a magnification of ×5000. Three glomeruli from each animal (*N* = 8 per group) were examined. In each animal (*N* = 8 per group), we also acquired five random images of cross-sections of the glomerular capillary wall, 10 cross-sectioned proximal tubules, 10 cross-sectioned distal tubules, and 10 cross-sectioned collecting ducts at a magnification of ×40,000. Further, we used these transmission electron microscopy (TEM) pictures to measure the width of the basement membranes of these structures.

#### 4.3.3. Morphometric Analysis

Morphometric analysis was performed with ImageJ software version 1.54p (Madison, WI, USA). We applied the Grid and Cell Counter plug-ins for the morphometric analysis of the images, as described below. The measurements were based on previously described methods [125].

The entire visual field area of the kidney section was used to assess the *V_V_* of glomeruli, proximal and distal tubules. The *V_V_* of these renal structures was determined by point counting with the use of a lattice square test system with a distance between lines of 5 mm, corresponding to 5 μm. Approximately 300 points were counted per region of each image. The *V_V_* values were calculated using the equation:

(1) $$V_{V}=\frac{N_{S}}{N_{T}}\times 100\%$$

where *V_V_* is volumetric density, *N_S_* is the number of points overlaid on the structure, *N_T_* is the total number of points.

We counted dots corresponding to mesangial cells, the mesangial matrix, and glomeruli to calculate MFV. We used a lattice square test system with a distance between lines of 4 mm, corresponding to 1 μm. Up to 450 points were counted per glomerulus in each image. The region of interest included the glomerular capillary tuft and excluded Bowman’s space, Bowman’s capsule, adjacent tubules, interstitium, and periglomerular vessels. MFV was determined by the equation:

(2) $$MFV=\frac{N_{M}}{N_{G}}\times 100\%$$

where *N_M_* is the number of points overlaid on the mesangial cells and matrix in the glomerulus and *N_G_* is the total number of points in one glomerulus.

In order to assess podocyte FP effacement, we measured the *N_L_* of podocyte FPs. We counted the number of podocyte FPs on the GBM segment in TEM images. The length of the GBM segment was measured with the selection tools of ImageJ software (Madison, WI, USA), such as straight and segmented lines. The 1 μm standard bar was applied for the measurements. We used the following equation to estimate the *N_L_* of podocyte FPs:

(3) $$N_{L}=\frac{N_{FPs}}{L}$$

where *N_L_* is a linear number density, *N*_FPs_ is the number of podocyte foot processes in a GBM section, and *L* is the length of a GBM section in µm.

#### 4.3.4. Measurements of Epithelial Cell Height and Basement Membrane Width

We used ImageJ software version 1.54p (Madison, WI, USA) to measure epithelial cell height and basement membrane width. We utilized selection tools such as straight lines to calculate values in physical units (μm for light microscopy and nm for TEM) by establishing an image scale.

On the light micrographs of the renal cortex and medulla that were acquired at a magnification of ×400, ten random cross-sections per animal were identified from each of the following nephron segments: proximal tubules, distal tubules, and collecting ducts. Then, we selected five random epithelial cells in each cross-section. In total, at least 50 epithelial cells of proximal tubules, distal tubules, and collecting ducts per animal (*N* = 8 in each group) were included in the analysis. We applied the 10 μm standard bar to assess the height of epithelial cells from the basement membrane to the luminal surface.

We measured the GBM and TBM widths by a direct method [126]. For this, we drew uniform cross-sections across the membrane and measured the exact pixel distance between the basal aspect of the epithelial membrane and the opposite edge. The measurements were carried out at intervals of at least 300 nm from each other. Five to ten measurements were performed in each image. The arithmetic mean of multiple evenly spaced measurements was then calculated for each animal (*N* = 8 per group). The 200 nm standard bar was applied for measurements.

#### 4.3.5. Processing of Morphometry Data

In the light microscopy images, we assessed 14 kidney sections, 112 glomeruli, 112 cross-sectioned proximal tubules, 112 distal tubules, and 112 collecting ducts in each group. We assessed the height of at least 560 PTECs, 560 DTECs and 560 CDECs in each group. In the TEM images, we analyzed 24 glomeruli, 40 glomerular barrier cross-sections, 80 proximal tubule cross-sections, 80 distal tubule cross-sections, and 80 collecting duct cross-sections in each group. We evaluated 280 GMB measurement points, 480 TBM measurement points, and 480 collecting duct basement membrane measurement points per group. We estimated 600 FPs in vehicle-treated *db/db* mice and 800 FPs in other groups. We then calculated the average values for each individual animal. These biological replicates were used as experimental units in the following statistical procedures.

### 4.4. Protein Extraction, WB and Multiplex Bead Array Assay

#### 4.4.1. Protein Extraction

Frozen samples of the renal cortex and renal medulla (*N* = 8) obtained from *db/+* and *db/db* mice were homogenized in radioimmunoprecipitation assay (RIPA) protein lysis buffer (50 mM Tris-HCl pH 8.0, 150 mM NaCl, 1% of Triton X-100, 1% of sodium deoxycholate, 0.1% of sodium dodecyl sulfate [SDS], and 1 mM ethylenediaminetetraacetic acid [EDTA]) with the proteinase inhibitor Proteinsafe (Transgene Biotech, Beijing, China). Protein extracts were stored at −20 °C for further investigations.

#### 4.4.2. WB

We randomly selected five of the eight animals in each group for WB. Equal amounts of protein were analyzed by immunoblot assays using antibodies to Beclin-1, LC3B, caspase-3 p17 subunit, Bcl-2, E-cadherin, N-cadherin, AMPK α1 and PI3Kp110β (Abcam, Cambridge, UK; ab207612, ab192890, ab13847, ab182858, ab40772, ab76011, ab32047, and ab151549, respectively). Anti-GAPDH antibody (Abcam, Cambridge, UK; ab9485) was used as a loading control. Intensities of individual bands were quantified by using ImageJ software version 1.54p (Madison, WI, USA).

#### 4.4.3. Normalization of WB Results

Taking into account possible changes in GAPDH under hyperglycemic conditions, we verified stable GAPDH expression across experimental groups. Firstly, we re-analyzed the density of the GAPDH bands. The Kruskal–Wallis test revealed no significant intergroup differences in GAPDH levels (*p* = 0.7 for renal cortex and *p* = 0.69 for renal medulla, Table S2, Figure S1), ruling out any systematic bias.

Secondly, we compared GAPDH expression stability with that of an alternative housekeeping protein (β-actin, Abcam, Cambridge, UK, ab6276) under our experimental conditions. We conducted an additional Western blot experiment with the protein extracts of the renal cortex and renal medulla (*N* = 5 per group). No differences in the signal intensity for β-actin were detected between samples from the studied groups in the renal cortex or medulla (*p* = 0.41 and *p* = 0.47, respectively, Table S2, Figure S1).

Finally, we analyzed the signal intensity for GAPDH and β-actin bands, as well as total protein by Ponceau S staining (Table S3; Figure S1). The normalization results for GAPDH, β-actin, and Ponceau S staining were compared. We found no difference between the intensities of the parameters (coefficient of variation for GAPDH < 5.0%).

#### 4.4.4. Multiplex Bead Array Assay

The concentrations of phosphorylated forms of some key molecules, regulators, and downstream targets of the PI3K/Akt pathway were assessed with the use of the Bio-Plex Pro™ Cell Signaling Akt Panel (catalog no. LQ00006JK0K0RR, Bio-Rad Laboratories, Hercules, CA, USA). This panel included BAD phosphorylated at Ser136 [phospho-BAD (Ser136)], GSK-3α/β phosphorylated at Ser21/Ser9 [phospho-GSK-3α (Ser21)/ p-GSK-3β (Ser9)], IRS-1 phosphorylated at Ser636/Ser639 [phospho-IRS-1 (Ser636/Ser639)], mTOR phosphorylated at Ser2248 [phospho-mTOR (Ser2248)], PTEN phosphorylated at Ser380 [phospho-PTEN (Ser380)], and rpS6 phosphorylated at Ser235/Ser236 [phospho-rpS6 (Ser235/236)]. The concentrations of some core components of the MAPK cascade and downstream targets of MAPK signaling were assessed with the use of the Bio-Plex Pro™ Cell Signaling MAPK Panel (catalog no. LQ00000S6KL81S, Bio-Rad Laboratories, Hercules, CA, USA). This kit was used to assess ERK1/2 phosphorylated at Thr202/Tyr204 and Thr185/Tyr187 [phospho-ERK1/2 (Thr202/Tyr204, Thr185/Tyr187)], MEK1 phosphorylated at Ser217/Ser221 [phospho-MEK1 (Ser217/Ser221)], HSP27 phosphorylated at Ser78 [phospho-HSP27 (Ser78)], p90^RSK^ phosphorylated at Ser15 [phospho-p90^RSK^ (Ser15)], and p53 phosphorylated at Ser380 [phospho-p53 (Ser380)].

The multiplex bead array assay was performed according to the manufacturer’s instructions. Renal protein extract samples in equal amounts of protein were incubated with antibody-coupled beads, detection antibody, and streptavidin for 30 min, 30 min, and 10 min, respectively. After each coating with antigen, the plate was washed with a Bio-PlexHandheld Magnetic Washer (Bio-Rad Laboratories, Hercules, CA, USA), resuspended and vortexed. Fluorescence was measured on a two-beam laser automated analyzer, the Bio-Plex^®^ 200 system. Data were acquired with Bio-PlexManager Software 4.0 (Bio-Rad Laboratories, Hercules, CA, USA). The values below the detection limit (OOR<) were set to zero. To validate the results, the positive and negative controls supplied with the kits were used.

Multiplex analysis was carried out in one technical replicate from one animal. We performed the assay with the Bio-Plex Pro™ Cell Signaling Akt Panel for eight animals per group. The assay, with the use of the Bio-Plex Pro™ Cell Signaling MAPK Panel, was performed on seven animals from each group.

### 4.5. Statistical Analysis

We considered data obtained from each individual animal as an experimental unit for statistical analysis. If several technical replicates were performed, the averaged results were used.

An outlier criterion was specified before examining group differences. Outliers were excluded using Grubbs’ test. Any data points with a two-sided *p*-value of less than 0.05 were considered significant outliers and were subsequently excluded from the analysis. The individual animal was considered the biological experimental unit for this analysis.

Statistical processing was performed using STATISTICA 12 (Dell, Round Rock, TX, USA). The normal distribution was determined by the Kolmogorov–Smirnov test. As most of the variables were not distributed normally, the data are presented as medians, minimum and maximum values, and the significance of the differences between independent groups was determined using the non-parametric Mann–Whitney U-test. The non-parametric Wilcoxon test was applied to compare dependent samples. Spearman’s rank correlation analysis was applied to test the association between variables. The differences were considered significant at *p* below 0.05. Correlation coefficients were considered significant if their absolute value was equal to or greater than 0.3. Due to the exploratory nature of the analysis, we did not apply multiple-comparison correction.

The power of the study was assessed by G*Power 3.1.9.4 (Dusseldorf, Germany) post hoc and ranged from 70% (for WB) to 80% (for multiplex bead array assays).

ANCOVA was carried out using SPSS Statistics 26.0 (IBM, Armonk, New York, NY, USA). The studied drugs were assumed to be a categorical independent variable, while baseline body weight and glucose levels were used as covariates. Associations were considered significant if the *p*-value was below 0.05. Only *db/db* mice, but not control animals, were included in this analysis.

### 4.6. Ethical Issues

All experiments were performed in compliance with the protocols and recommendations for the proper use and care of laboratory animals (Directive 2010/63/EU). The protocol was approved by the Inter-Institutional Animal Ethics Committee based on the Institute of Cytology and Genetics SB RAS (Protocol 72 dated 5 April 2021).

## 5. Conclusions

The results of the study demonstrate that the development of DKD in *db/db* mice, a model of T2D, is associated with dysregulation of the PI3K/Akt and MAPK/ERK signaling pathways in the kidneys. Particularly, these changes are characterized by decreased levels of PI3Kp110β and increased levels of phosphorylated PTEN, MEK1 and HSP27 in the renal cortex and medulla. Additionally, there is an increase in phosphorylated BAD and ERK1/2 in the renal cortex, as well as a decrease in rpS6 in the renal medulla.

Empagliflozin, a SGLT2 inhibitor, linagliptin, a DPP4 inhibitor, and metformin may have different effects on the studied signaling pathways. Specifically, empagliflozin prevented the changes in the levels of cortical PI3Kp110β, phospho-MEK1, and medullar phospho-PTEN; linagliptin restored PI3Kp110β levels; and both agents further decreased medullar phospho-rpS6. Metformin upregulated cortical AMPKα1, medullar PI3Kp110β, phospho-GSK-3α/β and MEK1 and increased phospho-HSP27 in the renal cortex and medulla. The changes in the PI3K/Akt and MAPK/ERK signaling pathways under empagliflozin, linagliptin and metformin treatment may contribute to the mitigation of albuminuria, renal hypertrophy, glomerular sclerosis and tubular atrophy. Direct effects on apoptosis, autophagy, EMT and fibrosis deserve further investigation.

## Figures and Tables

**Table 1:** Body weight and laboratory parameters of non-diabetic *db/+* mice and diabetic *db/db* mice treated by vehicle, empagliflozin, linagliptin or metformin.

Week of the Experiment	*db/+* Group, *N* = 8		*db/db* Groups		
		Vehicle, *N* = 8	Empagliflozin, *N* = 8	Linagliptin, *N* = 8	Metformin, *N* = 8
Body weight, g					
0	22.4 (21.9–23.4)	30.7 (23–33.1) ***	33.5 (26.1–38.3) ***	31.3 (25.7–36.2) ***	28 (23.9–32.4) **
4	26.5 (25.3–41.1) ^++^	37.9 (28.6–38.4) ***^++^	38.1 (33.3–38.8) ***^++^	38.2 (32.4–42.3) ***^++^	32.9 (28.3–34.7) ***^#^^^‡^
8	27 (25.65–27.75) ^++^	40.2 (26.7–42.6) ***^+++^	40.3 (38.5–40.6) ***^+++^	39.2 (33.6–44.3) ***^+++^	29.7 (27–34.7) **^#^^^‡^
Blood glucose, mmol/L					
0	5.8 (4.9–6.8)	11.8 (7.2–16.7) ***	16.7 (14–25.7) ***	18.9 (13–25.7) ***	13.5 (8.6–20.4) ***
4	6.3 (5–7.4)	24.1 (20.9–33.1) ***^++^	17.2 (9.8–26.3) ***	24.4 (22–33.1) ***^^++^	15.9 (12.7–23.3) ***^#‡+^
8	6.6 (5.6 –7.5)	22.2 (18.6–33.3) ***^+++^	16.7 (9.4–21.1) ***^#^	26.7 (21.2–33.3) ***^^+++^	17.3 (14–23.3) ***^#‡+^
Plasma fructosamine, µmol/L					
0	232 (213–243)	453 (422–516) ***	442 (407–585) ***	448 (367–574) ***	438 (402–582) ***
8	234 (221–286)	616 (512–654) ***	462 (320–498) ***^#‡^	625 (511–682) ***^	460 (322–584) ***^#‡^
Plasma creatinine, µmol/L					
0	64.4 (44.2–78.5)	73.6 (54.2–86.1)	70.2 (52.6–86.3)	72.6 (53.7–90.2)	70.8 (56.9–88.2)
8	62.3 (52.4–79.9)	76.2 (61.8–89.2) **	71.8 (55.8–91.2) *	74.4 (57.6–93.5) *	72.6 (55.6–89.4) **
Urinary albumin-to-creatinine ratio, mg/mmol					
0	0.40 (0.01–1.50)	12.5 (8.2–20.4) ***	14.2 (11.4–21.8) ***	16.4 (9.4–24.7) ***	13.6 (10.2–21.9) ***
8	0.50 (0.02–1.60)	11.4 (7.4–21.8) ***	6.4 (2.40–11.2) ***^###+++^	5.8 (3.4–12.7) ***^###+++^	12.8 (8.5–18.9) ***^^^^‡‡‡^
Kidney weight, g					
8	0.49 (0.46–0.53)	0.52 (0.45–0.56) *	0.48 (0.37–0.56)	0.47 (0.41–0.49)	0.43 (0.42–0.5)
Kidney weight/body weight, g/g					
8	0.019 (0.018–0.019)	0.014 (0.012–0.021)	0.013 (0.011–0.014) ^#^	0.012 (0.011–0.012) ^#^	0.014 (0.01–0.017)

* *p* < 0.05, ** *p* < 0.01, and *** *p* < 0.001 vs. control (*db/+* mice); ^#^ *p* < 0.05 and ^###^ *p* < 0.001 vs. vehicle-treated *db/db* mice; ^ *p* < 0.05 and ^^^ *p* < 0.001 vs. empagliflozin-treated *db/db* mice; ^‡^ *p* < 0.05 and ^‡‡‡^ *p* < 0.001 vs. linagliptin-treated *db/db* mice (Mann–Whitney test); ^+^ *p* < 0.05, ^++^ *p* < 0.01, and ^+++^ *p* < 0.001 vs. Week 0 (Wilcoxon test). The data are presented as medians, minimum and maximum values.

## Data Availability

The original contributions presented in this study are included in the article/Supplementary Materials. Further inquiries can be directed to the corresponding author.

## Supplementary Materials

The following supporting information can be downloaded at https://www.mdpi.com/article/10.3390/ijms27146483/s1.

## Abbreviations

The following abbreviations are used in this manuscript:

AMPK	5′-Adenosine monophosphate-activated protein kinase
AMPKα1	5′-Adenosine monophosphate-activated protein kinase catalytic subunit α1
ANCOVA	Analysis of covariance
BAD	Bcl-2-associated death promoter
Bcl-2	B-cell lymphoma 2
BM	Basement membrane
CARMELINA	The Cardiovascular and Renal Microvascular Outcome Study With Linagliptin
CDs	Collecting ducts
CKD	Chronic kidney disease
DTEC	Distal tubular epithelial cell
DPP4	Dipeptidyl peptidase-4
DKD	Diabetic kidney disease
EDTA	Ethylenediaminetetraacetic acid
ELISA	Enzyme-linked immunosorbent assay
EMPA-KIDNEY	The Study of Heart and Kidney Protection With Empagliflozin
EMPA-REG OUTCOME	The Empagliflozin Cardiovascular Outcome Event Trial in Type 2 Diabetes Mellitus Patients
EMT	Epithelial–mesenchymal transition
ERK	Extracellular signal-regulated kinase
ESRD	End-stage renal disease
FPs	Foot processes
HSP27	Heat shock protein 27
GAPDH	Glyceraldehyde 3-phosphate dehydrogenase
GBM	Glomerular basement membrane
GSK-3	Glycogen synthase kinase 3
IRS-1	Insulin receptor substrate 1
LC3B	Microtubule-associated proteins 1A/1B light chain 3B
LC3-I	Microtubule-associated proteins 1A/1B light chain 3B cytosolic form
LC3-II	Microtubule-associated proteins 1A/1B light chain 3B lipidated form
MAPK	Mitogen-activated protein kinase
MEK1	Dual specificity mitogen-activated protein kinase kinase 1
MFV	Mesangial fractional volume
mTOR	Mammalian target of rapamycin
*N_L_*	Lineal number density
p90^RSK^	Subfamily p90 ribosomal s6 kinase
PI3K/Akt	Phosphatidylinositol 3 kinases/protein kinase B
PI3Kp110β	Phosphoinositide 3-kinase p110β catalytic subunit β isoform
PTEC	Proximal tubular epithelial cell
PTEN	Phosphatase and tensin homolog
rpS6	Ribosomal protein S6
RSK	Ribosomal s6 kinase
SGLT2	Sodium/glucose cotransporter 2
SDS	Sodium dodecyl sulfate
T2D	Type 2 diabetes
TBM	Tubular basal membrane
TEM	Transmission electron microscopy
TGFβ1	Transforming growth factor-β1
*V_V_*	Volume fraction
WB	Western blotting
