# Supplementary material for: Empagliflozin, Linagliptin, and Metformin Differentially Affect Renal PI3K/Akt and MAPK/ERK Signaling Pathways in db/db Diabetic Mice
Source: Int J Mol Sci. 2026 Jul 21;27(14):6483. doi: 10.3390/ijms27146483 (PMC13409939; doi:10.3390/ijms27146483)
Supplement: Supplementary file 1 [file ijms-27-06483-s001.zip › ijms-4321677-supplementary.pdf]

Table S1. Associations of baseline body weight and blood glucose with the levels of PI3K/Akt and MAPK/ERK signaling mediators, autophagy-related, apoptosis-related and EMT-related markers in the kidney across *db/db* mice treated by vehicle, empagliflozin, linagliptin or metformin.

Dependent variable	Drug			Covariate		Adjusted R^2
	Empagliflozin	Linagliptin	Metformin	Baseline body weight	Baseline blood glucose	
Renal cortex						
AMPKα1	F=0.45, p=0.52, η²=0.04	F=17.59, p=0.002, η²=0.62	F=18.84, p=0.001, η²=0.63	F=0.92, p=0.36, η²=0.08	F=4.56, p=0.06, η²=0.29	0.66
PI3Kp110β	F=9.18, p=0.01, η²=0.46	F=6.98, p=0.02, η²=0.39	F=2.03, p=0.18, η²=0.16	F=0.21, p=0.66, η²=0.02	F=0.02, p=0.88, η²=0.002	0.3
E-cadherin	F=6.45, p=0.03, η²=0.37	F=2.02, p=0.18, η²=0.16	F=9.53, p=0.01, η²=0.46	F=0.12, p=0.74, η²=0.01	F=0.45, p=0.51, η²=0.04	0.39
N-cadherin	F=80.7, p<0.001, η²=0.88	F=22.1, p=0.001, η²=0.67	F=37.5, p<0.001, η²=0.77	F=0.01, p=0.91, η²=0.001	F=0.02, p=0.9, η²=0.002	0.89
p-BAD	F=0.05, p=0.83, η²=0.004	F=0.003, p=0.95, η²=0.0003	F=0.206, p=0.66, η²=0.02	F=8.27, **p=0.01**, η²=0.41	F=0.202, p=0.66, η²=0.02	0.21
p-GSK-3α/β	F=0.099, p=0.76, η²=0.008	F=0.155, p=0.7, η²=0.01	F=0.402, p=0.54, η²=0.03	F=0.419, p=0.53, η²=0.03	F=4.28, p=0.06, η²=0.26	0.05
p-IRS-1	F=0.047, p=0.83, η²=0.004	F=0.873, p=0.37, η²=0.07	F=2.81, p=0.12, η²=0.19	F=0.023, p=0.88, η²=0.002	F=2.45, p=0.14, η²=0.17	0.02
p-mTOR	F=1.72, p=0.21, η²=0.13	F=0.002, p=0.96, η²=0.0002	F=0.048, p=0.83, η²=0.004	F=0.671, p=0.43, η²=0.05	F=0.037, p=0.85, η²=0.003	0.06
p-PTEN	F=0.416, p=0.53, η²=0.03	F=0.003, p=0.96, η²=0.0002	F=1.27, p=0.28, η²=0.096	F=6.18, **p=0.03,** η²=0.34	F=0.568, p=0.47, η²=0.05	0.15
p-rpS6	F=0.51, p=0.49, η²=0.04	F=0.11, p=0.74, η²=0.009	F=0.63, p=0.44, η²=0.05	F=0.001, p=0.97, η²=0.0001	F=0.045, p=0.84, η²=0.004	0.33
p-ERK1/2	F=2.39, p=0.15, η²=0.19	F=2.23, p=0.17, η²=0.18	F=4.09, p=0.07, η²=0.29	F=4.88, p=0.052, η²=0.33	F=4.14, p=0.07, η²=0.29	0.11
p-MEK1	F=0.221, p=0.65, η²=0.02	F=0.653, p=0.44, η²=0.06	F=4.91, p=0.051, η²=0.33	F=2.54, p=0.14, η²=0.2	F=5.64, **p=0.04,** η²=0.36	0.32
p-HSP27	F=7.93, p=0.02, η²=0.44	F=5.24, p=0.045, η²=0.34	F=28.6, p<0.001, η²=0.74	F=22.4, **p=0.0008,** η²=0.69	F=19.9, **p=0.001,** η²=0.67	0.73

phospho-p53	F=0.366, p=0.56, η^2=0.04	F=0.394, p=0.54, η^2=0.04	F=0.002, p=0.97, η^2=0.0002	F=0.537, p=0.48, η^2=0.05	F=0.027, p=0.87, η^2=0.003	0.28
phospho-p90^{RSK}	F=3.82, p=0.08, η^2=0.28	F=0.983, p=0.34, η^2=0.09	F=1.31, p=0.28, η^2=0.12	F=1.52, p=0.25, η^2=0.13	F=4.29, p=0.07, η^2=0.3	0.05
Renal medulla						
AMPKα1	F=1.65, p=0.22, η^2=0.12	F=2.36, p=0.15, η^2=0.16	F=1.73, p=0.21, η^2=0.13	F=1.89, p=0.19, η^2=0.14	F=0.98, p=0.34, η^2=0.08	0.38
PI3Kp110β	F=0.4, p=0.54, η^2=0.03	F=3.13, p=0.1, η^2=0.21	F=6.07, p=0.03, η^2=0.34	F=1.61, p=0.23, η^2=0.12	F=0.79, p=0.39, η^2=0.06	0.34
E-cadherin	F=26.5, p<0.001, η^2=0.69	F=7.16, p=0.02, η^2=0.37	F=12.3, p=0.004, η^2=0.51	F=0.07, p=0.79, η^2=0.006	F=0.13, p=0.73, η^2=0.01	0.71
N-cadherin	F=81.9, p<0.001, η^2=0.87	F=19, p=0.001, η^2=0.61	F=108, p<0.001, η^2=0.9	F=1.21, p=0.29, η^2=0.09	F=0.18, p=0.68, η^2=0.02	0.91
Beclin-1	F=3.65, p=0.09, η^2=0.29	F=2.64, p=0.14, η^2=0.23	F=53.5, p<0.001, η^2=0.86	F=0.33, p=0.58, η^2=0.04	F=1.38, p=0.27, η^2=0.13	0.9
LC3-II	F=16, p=0.003, η^2=0.64	F=55.9, p<0.001, η^2=0.86	F=72.3, p<0.001, η^2=0.89	F=9.35, **p=0.01**, η^2=0.51	F=5.04, p=0.051, η^2=0.36	0.88
Caspase-3 p17 subunit	F=45.6, p<0.001, η^2=0.84	F=10.8, p=0.009, η^2=0.55	F=46.8, p<0.001, η^2=0.84	F=1.57, p=0.24, η^2=0.15	F=0.69, p=0.43, η^2=0.07	0.81
Bcl-2	F=61.9, p<0.001, η^2=0.87	F=61.1, p<0.001, η^2=0.87	F=11.2, p=0.009, η^2=0.56	F=0.16, p=0.7, η^2=0.02	F=0.0001, p=0.99, η^2=0.00001	0.86
p-BAD	F=0.784, p=0.39, η^2=0.06	F=2.76, p=0.12, η^2=0.19	F=0.134, p=0.72, η^2=0.01	F=8.01, **p=0.02,** η^2=0.4	F=8.14, **p=0.01,** η^2=0.4	0.47
p-GSK-3α/β	F=2.02, p=0.18, η^2=0.14	F=1.35, p=0.27, η^2=0.1	F=0.169, p=0.69, η^2=0.01	F=2.05, p=0.18, η^2=0.15	F=0.521, p=0.48, η^2=0.04	0.22
p-IRS-1	F=5.1, p=0.04, η^2=0.3	F=4.42, p=0.06, η^2=0.27	F=0.014, p=0.91, η^2=0.001	F=9.8, **p=0.009**, η^2=0.45	F=1.59, p=0.23, η^2=0.12	0.59
p-mTOR	F=0.878, p=0.37, η^2=0.07	F=2.32, p=0.15, η^2=0.16	F=0.235, p=0.64, η^2=0.02	F=0.022, p=0.89, η^2=0.002	F=0.556, p=0.47, η^2=0.04	0.14
p-PTEN	F=3.15, p=0.1, η^2=0.21	F=1.35, p=0.27, η^2=0.1	F=1.26, p=0.28, η^2=0.09	F=0.493, p=0.5, η^2=0.04	F=0.471, p=0.51, η^2=0.04	0.09
p-rpS6	F=0.48, p=0.5, η^2=0.04	F=0.4, p=0.54, η^2=0.03	F=0.27, p=0.61, η^2=0.02	F=0.273, p=0.61, η^2=0.02	F=0.061, p=0.81, η^2=0.005	0.34

p-ERK1/2	F=1.52, p=0.25, η^2=0.13	F=0.359, p=0.56, η^2=0.03	F=1.86, p=0.2, η^2=0.16	F=1.23, p=0.29, η^2=0.11	F=0.002, p=0.97, η^2=0.0002	0.12
p-MEK1	F=0.043, p=0.84, η^2=0.004	F=0.155, p=0.7, η^2=0.02	F=0.084, p=0.78, η^2=0.008	F=0.89, p=0.37, η^2=0.08	F=0.68, p=0.43, η^2=0.06	0.16
p-HSP27	F=1.85, p=0.2, η^2=0.16	F=0.011, p=0.92, η^2=0.001	F=0.08, p=0.79, η^2=0.008	F=1.91, p=0.2, η^2=0.16	F=0.165, p=0.69, η^2=0.02	0.19
phospho-p53	F=7.31, p=0.02, η^2=0.422	F=3.82, p=0.08, η^2=0.28	F=5.82, p=0.04, η^2=0.368	F=0.142, p=0.71, η^2=0.01	F=1.18, p=0.3, η^2=0.11	0.24
phospho-$p90^{RSK}$	F=3.39, p=0.1, η^2=0.25	F=0.073, p=0.79, η^2=0.007	F=1.72, p=0.22, η^2=0.15	F=3.05, p=0.11, η^2=0.23	F=0.458, p=0.51, η^2=0.04	0.42

The results of analysis of covariance (ANCOVA) with F-statistics, p-values, partial η^2 are presented. Treatment by empagliflozin, linagliptin or metformin is assumed as categorical independent variable. R^2, coefficient of determination. Other abbreviations are explained into the main text of the article. p-values below 0.05 for baseline body weight and blood glucose are highlighted in bold.

Table S2. Western blot analysis of GAPDH and β-actin signal intensity in non-diabetic *db*/+ mice and diabetic *db*/*db* mice treated by vehicle, empagliflozin, linagliptin or metformin.

**Loading control**	***db*/+ mice** N = 5	***db*/*db* mice** **Vehicle** N = 5	**Empagliflozin** N = 5	**Linagliptin** N = 5	**Metformin** N = 5	Kruskal–Wallis test
			Renal cortex			
GAPDH	29048 (23665–32057)	27846 (22861–30059)	25996 (24886–26806)	26498 (24644–27722)	25492 (21919–30118)	H = 2.18, df = 4, p = 0.7
β-actin	25788 (24733–33498)	31244 (16549–31700)	24941 (14986–31231)	25494 (16648–32331)	22790 (17431–35192)	H = 3.97, df = 4, p = 0.41
			Renal medulla			
GAPDH	27674 (23167–32939)	29711 (23381–31971)	29767 (25587–31263)	28213 (23629–32370)	25153 (22714–30930)	H = 2.24, df = 4, p = 0.69
β-actin	38897 (36017–39302)	39049 (32030–46321)	38514 (36026–47087)	38897 (34310–46686)	39713 (35203–46321)	H = 3.55, df = 4, p = 0.47

The data are presented as medians (minimal – maximal values). df, degrees of freedom; H, the test statistic for the Kruskal-Wallis test; GAPDH, glyceraldehyde 3-phosphate dehydrogenase.

Table S3. Western blot analysis of GAPDH and β-actin signal intensity normalized to total protein by Ponceau S staining in non-diabetic *db*/+ mice and diabetic *db*/*db* mice treated by vehicle, empagliflozin, linagliptin, or metformin.

Parameter	*db*/+ mice	*db*/*db* mice				All groups
		Vehicle	Empagliflozin	Linagliptin	Metformin	
GAPDH / total protein	0.04856	0.04834	0.04718	0.04748	0.04809	0.04813
GAPDH / total protein, SD	0.00029	0.00074	0.00101	0.00173	0.00048	0.00114
GAPDH / total protein, CV	0.6	1.54	2.14	3.64	0.99	2.37
β-actin / total protein	0.04874	0.04812	0.04876	0.05011	0.04842	0.04883
β-actin / total protein, SD	0.00237	0.00152	0.00253	0.00054	0.00005	0.00146
β-actin / total protein, CV	4.87	3.15	5.19	1.08	0.1	3

CV, coefficient of variation, %; SD, standard deviation; GAPDH, glyceraldehyde 3-phosphate dehydrogenase.

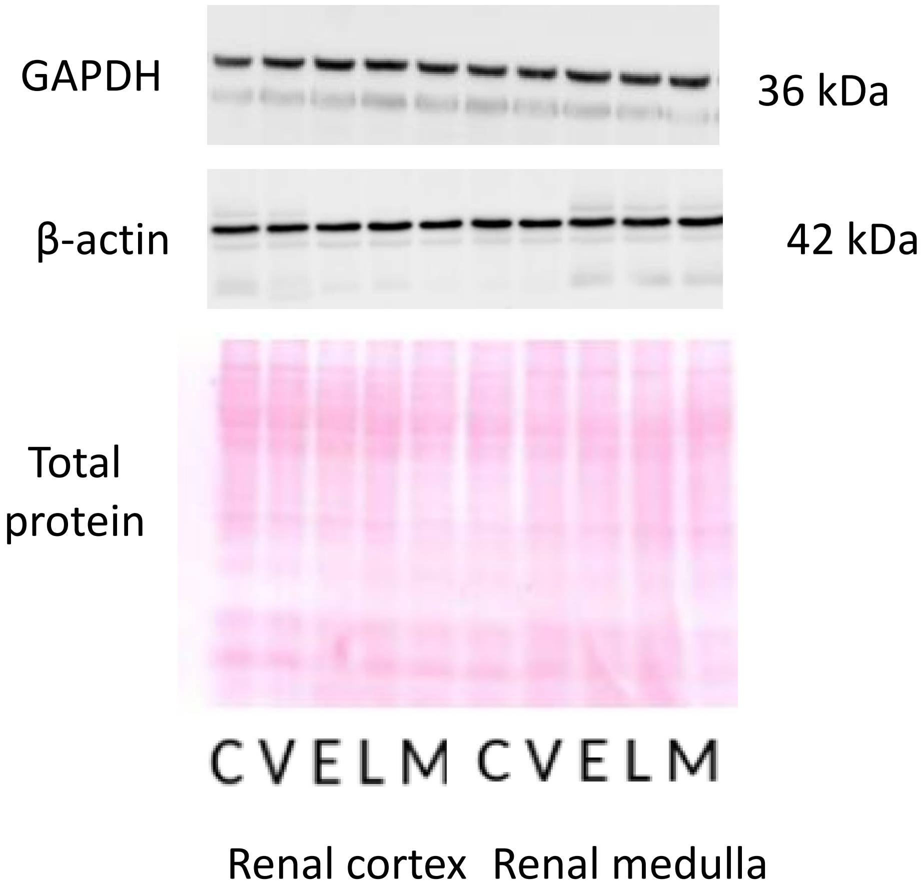

Figure S1. Representative images of Western blot for β-actin, GAPDH, and total protein by Ponceau S staining. C, non-diabetic *db*/+ mice; E, empagliflozin-treated *db*/*db* mice; L, linagliptin-treated *db*/*db* mice; M, metformin-treated *db*/*db* mice; V, vehicle-treated *db*/*db* mice; GAPDH, glyceraldehyde 3-phosphate dehydrogenase.
